# Deciphering immune responses primed by a bacterial lipopeptide in wheat towards *Zymoseptoria tritici*

**DOI:** 10.3389/fpls.2022.1074447

**Published:** 2023-01-26

**Authors:** Rémi Platel, Anca Lucau-Danila, Raymonde Baltenweck, Alessandra Maia-Grondard, Pauline Trapet, Maryline Magnin-Robert, Béatrice Randoux, Morgane Duret, Patrice Halama, Jean-Louis Hilbert, François Coutte, Philippe Jacques, Philippe Hugueney, Philippe Reignault, Ali Siah

**Affiliations:** ^1^ Joint Research Unit 1158 BioEcoAgro, Junia, Université de Lille, Université de Liège, UPJV, Université d’Artois, ULCO, INRAE, Lille, France; ^2^ Université de Strasbourg, INRAE SVQV UMR A1131, Colmar, France; ^3^ Unité de Chimie Environnementale et Interactions sur le Vivant, Université du Littoral Côte d’Opale, Calais Cedex, France; ^4^ Joint Research Unit 1158 BioEcoAgro, TERRA Teaching and Research Centre, MiPI, Gembloux Agro-Bio Tech, Université de Liège, Gembloux, Belgium

**Keywords:** wheat, *Zymoseptoria tritici*, induced resistance, priming, lipopeptide, omics

## Abstract

Plant immunity induction with natural biocontrol compounds is a valuable and promising ecofriendly tool that fits with sustainable agriculture and healthy food. Despite the agroeconomic significance of wheat, the mechanisms underlying its induced defense responses remain obscure. We reveal here, using combined transcriptomic, metabolomic and cytologic approach, that the lipopeptide mycosubtilin from the beneficial bacterium *Bacillus subtilis*, protects wheat against *Zymoseptoria tritici* through a dual mode of action (direct and indirect) and that the indirect one relies mainly on the priming rather than on the elicitation of plant defense-related mechanisms. Indeed, the molecule primes the expression of 80 genes associated with sixteen functional groups during the early stages of infection, as well as the accumulation of several flavonoids during the period preceding the fungal switch to the necrotrophic phase. Moreover, genes involved in abscisic acid (ABA) biosynthesis and ABA-associated signaling pathways are regulated, suggesting a role of this phytohormone in the indirect activity of mycosubtilin. The priming-based bioactivity of mycosubtilin against a biotic stress could result from an interaction of the molecule with leaf cell plasma membranes that may mimic an abiotic stress stimulus in wheat leaves. This study provides new insights into induced immunity in wheat and opens new perspectives for the use of mycosubtilin as a biocontrol compound against *Z. tritici*.

## 1 Introduction

Wheat is one of the most consumed cereal crops worldwide, serving as primary ingredient in human nutrition, food industry, and livestock feed. Wheat cultivation has to cope with a wide range of phytopathogenic microorganisms impacting its growth, productivity, and the quality of its production (Savary et al., 2019). The most frequently occurring and damaging pathogen on wheat crop is the hemibiotrophic fungus *Zymoseptoria tritici*, responsible for Septoria tritici blotch (STB) disease, causing severe yield losses of up to 50% during high-level pressure years (Fones and Gurr, 2015). The infection process of this hemibiotrophic pathogen consists of an initial asymptomatic biotrophic phase followed by a necrotrophic phase, the latter being characterized by the apparition of visual symptoms (Steinberg, 2015). Even if progresses have been accomplished in wheat resistance breeding during the last decades, with 22 major resistance genes described against *Z. tritici*, disease control of STB relies mainly on synthetic fungicides (Brown et al., 2015; Torriani et al., 2015; Yang et al., 2018). In Europe, 70% of the total currently applied fungicides are used to protect wheat against *Z. tritici* (Fones and Gurr, 2015). Nevertheless, fungicide resistance developed by *Z. tritici* populations is an increasing concern for wheat producers (Cools and Fraaije, 2013; McDonald et al., 2019). Therefore, a strong need for substitutes and alternatives have emerged in the recent years, reinforced by all the concerns about the potential impacts of chemical inputs on the environment and human health.

Biosurfactants are surface-active biomolecules produced by a broad range of microorganisms, including bacteria and fungi. These molecules are often classified according to their chemical structure, such as mannosylerythritol lipids, trehalose dimycolate, trehalolipids, sophorolipids, rhamnolipids, and lipopeptides (Crouzet et al., 2020). Even though they may be very diverse both in their structure and origin, they all are amphiphilic molecules, composed by hydrophilic and hydrophobic moieties. They also exhibit a high potential for application in many fields, including human health, cosmetic, food industry, petroleum industry, soil and water remediation, nanotechnology and agriculture (Sachdev and Cameotra, 2013; Shekhar et al., 2015; Kourmentza et al., 2021). Regarding their use in crop protection, many studies have reported them as promising ecofriendly candidates for biocontrol of crop diseases (Ongena and Jacques, 2008; D’aes et al., 2010; Sachdev and Cameotra, 2013; Crouzet et al., 2020). Moreover, green surfactants have often been described as displaying high biodegradability, as well as low toxicity and ecotoxicity, two major advantages required for the development of sustainable agriculture (Shekhar et al., 2015).

The possible modes of action of biosurfactants in plant protection against pathogens are diverse. For instance, they can display direct antimicrobial activity towards pathogens, modify the bio-availability of nutrients used by the pathogens, and/or induce plant immune defenses (D’aes et al., 2010). The compounds inducing the plant immune defenses may be distinguished into two categories, including elicitors, that directly activate host defense responses after their application, and priming agents, that require additional signals, such as pathogen recognition, to trigger full defense responses (Paré et al., 2005). Primed plants may develop a stronger and/or faster response pattern than so-called naïve (unprimed) plants. They can also detect the pathogen invasion at a lower threshold and hence react in a more sensitized way. Finally, primed plants can exhibit other response networks, involving specific defense pathways (Conrath et al., 2002; Paré et al., 2005; Lämke and Bäurle, 2017).

*Bacillus* spp. are considered as microbial factories for the production of metabolites of interest, especially biosurfactant molecules such as lipopeptides. Lipopeptides consist of a hydrophobic fatty acid tail linked to a short linear or cyclic oligopeptide (CLPs), and were, initially, mainly studied for their direct antimicrobial activities against phytopathogenic agents. However, many investigations have also been carried out on their stimulating effect of host defense mechanisms (Ongena and Jacques, 2008; Raaijmakers et al., 2010; Chowdhury et al., 2015; Crouzet et al., 2020). Three main groups of CLPs have been reported to exhibit significant biological activities, including surfactins, iturins and fengycins. The two latter possess a significant antimicrobial activity against a wide range of fungi and oomycetes, likely due to their ability to interact directly with pathogen plasma membranes, hence resulting in their destabilization, pore formation and cytoplasmic leakage, leading *in fine* to cell death or to an inhibition of spore germination (Chitarra et al., 2003; Pérez-García et al., 2011; Desmyttere et al., 2019; Crouzet et al., 2020). However, some authors suggest that CLPs could also display antifungal activity by interacting with fungal intracellular targets (Qi et al., 2010). Concerning their ability to induce plant immunity, many studies have highlighted the potential of mainly two families of CLPs, fengycin and surfactin, to trigger defense reactions in various plants, such as bean, citrus, grapevine, lettuce, tomato, melon, rye grass, sugar beet, and wheat, as reviewed by Crouzet et al. (2020). Mycosubtilin, another CLP was also shown to induce defense responses in grapevine cells (Farace et al., 2015). Supposedly, CLPs are able to fit into the lipid bilayer of host plant plasma membranes, slightly altering the lipid dynamics, hence leading to the activation of defense reactions, as eliciting or priming agents (Nasir et al., 2010; Schellenberger et al., 2019).

Although the innate immunity of wheat towards *Z. tritici* is extensively studied (*e.g.* Rudd et al., 2015; Seybold et al., 2020), the literature regarding the induced resistance in wheat against this major fungal pathogen, with exogenous treatments, is relatively poor. Regarding CLPs, only one single study showing the potential of surfactin to activate defense mechanisms in wheat against *Z. tritici* has been recently reported (Le Mire et al., 2018). Nevertheless, the ability of mycosubtilin, a CLP from the iturin family, to induce defense reactions in wheat against this disease has never been investigated, while its direct antimicrobial activity against *Z. tritici* has already been examined (Mejri et al., 2018). Omics tools, including transcriptomics and metabolomics, have significantly improved these later years the understanding of plant-pathogen cross-talks. Although such approaches have already been used to investigate the interactions between wheat and *Z. tritici*, they have not been deployed so far to examine the induced resistance in this pathosystem. The aim of the present study was thus to determine whether mycosubtilin is able to trigger immunity reactions in wheat towards *Z. tritici* as an eliciting (in absence of fungal infection) or a priming (in presence of fungal infection) molecule, by using transcriptomic and metabolomic tools. The analyses allowed to decipher the mechanisms as well as the plant-defense related pathways induced by the biomolecule. Cytological bioassays were also performed to better understand the mode of action of mycosubtilin on both the fungus and the host plant.

## 2 Materials and methods

### 2.1 Plant growth, treatment and inoculation

Seeds of the cultivar (cv.) Alixan (Limagrain, France), susceptible to STB, were pregerminated in square Petri dishes (12 × 12 cm) on moist filter paper as described by Siah et al. (2010a), and germinated grains were delicately transferred into three-liter pots filled with universal loam (Gamm Vert, France). The prepared pots were then placed in the greenhouse at 21 ± 2°C with a 16/8 h day-night cycle. For each condition and each sampling modality, three pots harboring 12 wheat plants each (n=36), were used. At day 0 (D0), corresponding to three weeks after sowing, plants of each pot were hand-sprayed with 30 mL of either a solution of 100 mg.L^-1^ mycosubtilin from *Bacillus subtilis* (Lipofabrik, Lesquin, France) or mock (control) treatment. Mycosubtilin was first dissolved in dimethyl sulfoxide (DMSO, Sigma-Aldrich, Saint-Louis, USA), leading to a final DMSO concentration of 0.1% in the treatment solution, also supplemented with 0.05% of polyoxyethylene-sorbitan monolaurate (Tween 20, Sigma-Aldrich, Saint-Louis, USA) serving as wetting agent. Mock treatment consisted of a solution of 0.1% DMSO supplemented with 0.05% of Tween 20. At two days after treatment (D2), half of the plants treated with mycosubtilin, as well as control plants, were inoculated by hand-spraying 30 mL of *Z. tritici* spore suspension (10^6^ spores.mL^-1^), mixed with 0.05% of Tween 20, on the plants of each pot. Fungal spores were obtained by growing the *Z. tritici* single-spore strain T02596 (isolated in 2014 in Northern France) on potato dextrose agar (PDA) medium during one week in dark conditions, according to Siah et al. (2010a). The other half of the plants were mock inoculated by hand spraying 30 mL of a 0.05% Tween 20 solution on the plants of each pot. All pots, even the mock inoculated ones, were then immediately covered with a clear polyethylene bag for three days in order to ensure a high humidity level on the surface of the leaves, a required condition for the success of the infection. At 23 days after treatment (D23), the disease severity level was assessed by scoring, on the plant third leaves, the area of lesions (chlorosis and necrosis) bearing or not pycnidia. Leaf samples were harvested at two (D2), five (D5) and fifteen (D15) days after treatment for further cytological, biochemical, or molecular analyses. In addition to the protection efficacy assay, a phytotoxicity biotest was performed in order to evaluate the impact of mycosubtilin at different concentrations (0, 0.8, 4, 20, 100, and 500 mg L^-1^) on leaf appearance and plant fresh biomass. The assay was carried out in the greenhouse using the same conditions of plant growth and treatment described above. Leaf appearance was assessed on the third leaves at two (D2), seven (D7), and 15 (D15) days after treatment, while the plant fresh biomass was determined at 15 days after treatment. Three pots of nine plants each (27 plants in total) were used as replicates for each molecule concentration condition.

### 2.2 *In vitro* antifungal activity assay in solid medium

The *in vitro* antifungal effect of mycosubtilin was determined by measuring the growth of *Z. tritici* strain T02596 on PDA medium, as described by Platel et al. (2021). Briefly, mycosubtilin was dissolved in DMSO (0.1% final DMSO concentration) and different concentrations of this biomolecule (0.19, 0.39, 0.78, 1.56, 3.125, 6.25, 12.5, 25, 50, and 100 mg.L^-1^) were mixed with an autoclaved PDA medium at approximatively 40°C. Each well of sterile 12-well plates (Cellstar standard^®^, Greiner Bio-One GmbH, Kremsmünster, Austria) was filled with 3 mL of the mixture. After PDA solidification, a drop of 5 µL of *Z. tritici* spore suspension at 5.10^5^ spores.mL^-1^ was spotted on the center of each plate well. After drop drying in sterile conditions, the plates were incubated in a growth chamber at 20 ± 1°C in dark conditions. Ten days later, the growth of fungal colonies was assessed by measuring their two perpendicular diameters. Control wells supplemented or not with 0.1% DMSO and inoculated or not with fungal spores were also used. This experiment was repeated twice and three wells were used as replicates for each condition.

### 2.3 *In vitro* direct activity bioassays in liquid medium

The *in vitro* direct antifungal activity of mycosubtilin in liquid medium was assessed by measuring the cell viability of *Z. tritici* strain T02596 using resazurin staining, as proposed by Siah et al. (2010b). Sterile 12-well plates were also used for the bioassay. Plate wells were filled with 2 mL of autoclaved glucose peptone medium supplemented with mycosubtilin at different concentrations (0.8, 4, 20, 100, and 500 mg.L^-1^), before inoculation with fungal spore suspension already prepared in sterile glucose peptone medium, for a final concentration of 5.10^4^ spores.mL^-1^. Mycosubtilin was first dissolved in DMSO at a final concentration of 0.5% in the wells. Controls with and without 0.5% DMSO and with or without the fungus, were also used. After incubation for ten days at 20 ± 1°C under agitation at 150 rpm in dark conditions, 140 µL of the content of each well were sampled and deposited in flat-bottomed polystyrene 96-well microplates (Corning Costar^®^, Corning, USA). Then, 10 µL Alamar blue (AbD Serotec, UK) were added to each well before incubating the microplates for 4h in the same conditions described above. Cell viability was evaluated by measuring the absorbance at 540 nm using a microplate reader (Multiskan GO, Thermo Fischer Scientific, France). Six biological replicates were used for each condition.

The inhibitory effect of mycosubtilin towards unique wheat cells (cv. Alixan) was assessed in sterile 12-well plates. Wheat unique cells were obtained using the protocol of Biesaga-Kościelniak et al. (2008), with few modifications. Briefly, wheat caryopses were plunged for 5 min in 70% ethanol, rinsed twice with sterile osmotic water, and disinfected for 20 min in a 15% calcium hypochlorite solution, before being cleansed again at least twice. Mature embryos were delicately separated from the rest of the grain and were gently ground in a mortar. Fifty embryos were required to initiate wheat-cell suspension in 200 mL Erlenmeyer flask. These flasks contained 20 mL of autoclaved Gamborg B5 medium including vitamins (Duchefa Biochemie B.V, Netherlands), supplemented with 20 g.L^-1^ sucrose and 3.5 mg.L^-1^ 2,4-dichlorophenoxyacetic acid (Thermo Fischer Scientific, France). Wheat cell suspensions were incubated at 20 ± 1°C in dark conditions under agitation at 80 rpm. At three and six days after inoculation, 20 mL of sterile medium were added to each flask. At 10 days, the mixture was filtered through a 1 mm sieve, to get rid of ungrounded agglomerate tissue. Fresh medium was added to the filtrates to obtain 80 mL suspension in each flask. Seven days later, 20 mL of Gamborg B5 fresh medium were supplemented. Finally, every week, 25% of the suspension was discarded and replaced by fresh medium. After two months of subculture, 12-well plate wells were filled with 2 mL of wheat cell suspension as well as 2 mL of fresh Gamborg B5 medium supplemented with mycosubtilin at different concentrations (0.8, 4, 20, 100, and 500 mg.L^-1^). The plates were then placed into a growth chamber at 20 ± 1°C in the dark with an agitation of 80 rpm. Three weeks after incubation, the number of wheat cells were counted for each condition using a Malassez hemocytometer under a light microscope (Nikon, Champigny‐sur‐Marne, France). Representative pictures were obtained using a digital camera (DXM1200C, Nikon, Champigny‐sur‐Marne, France) coupled with the image capture software NIS-Elements BR (Nikon, Champigny‐sur‐Marne, France).

### 2.4 *In planta* cytological assays

The direct antifungal activity of mycosubtilin at 100 mg.L^-1^ on *Z. tritici* spore germination as well as on the epiphytic hyphal growth of the fungus on the leaf surface was assessed in planta at D5 using Fluorescent Brightener 28 (Calcofluor, Sigma-Aldrich, Saint-Louis, USA), a chitin staining dye. Third-leaf segments (2 cm) harvested from wheat plants grown in the greenhouse, inoculated with *Z. tritici* and treated or not with mycosubtilin in the same conditions described above, were sampled and immersed for 5 min in a solution of 0.1% (w/v) Calcofluor buffered with 0.1 M Tris-HCl, pH 8.5. Leaf segments were then rinsed twice in sterile osmosed water for 2 min before being dried in dark conditions at room temperature. Finally, they were deposited on a glass slide, covered with cover slip, and observed with an optic microscope (Eclipse 80i, Nikon, Champigny‐sur‐Marne, France) under UV-light. The proportion of different spore classes was determined from 100 distinct spores on each third-leaf segment. Spore classes were defined as follows; class 1, non-germinated spore; class 2, germinated spore with a small germ tube; class 3, germinated spore with developed germ tube; and class 4, geminated spore with a strongly developed germ tube. Nine leaf segments, randomly selected from three different pots (three segments per pot), were used as replicates for each condition. Pictures were obtained using digital camera (DXM1200C, Nikon, Champigny‐sur‐Marne, France) coupled with the image capture software NIS-Elements BR (Nikon, Champigny‐sur‐Marne, France).

### 2.5 Sampling design for transcriptomic and metabolomic analyses

Samples were collected at two days (D2) and five days (D5) after treatment for transcriptomic analyses. For each condition, *i.e.* plants treated with mycosubtilin (M) or not (control, C), and inoculated with *Z. tritici* (i) or not (Ni), nine third leaves sampled from three different pots (three leaves per pot) were randomly harvested, bulked per pot, immediately frozen in liquid nitrogen and stored at -80°C for further analyses. Thereafter, 100 mg of frozen pooled leaves were ground in a mortar with liquid nitrogen for RNA extraction. Regarding metabolomic analyses, samples were collected at D2, D5, as well as fifteen days after treatment (D15). For each modality, nine third leaves were randomly harvested from three different pots (three leaves per pot), immediately frozen in liquid nitrogen, and stored at -80°C for further analyses. Leaves were later freeze-dried using Alpha 2-4 LSCplus lyophilizer (Martin Christ Gefriertrocknungsanlagen GmbH, Osterode am Harz, Germany) and ground with iron beads using a MM2 Retsch mixer-mill (Retsch GmbH, Haan, Germany) in order to obtain a fine powder, suitable for analyses. Sampling design for both transcriptomic and metabolomic analyses is illustrated in **Supplementary Figure S1**.

### 2.6 RNA extraction and microarray analyses

Total RNA was extracted from wheat leaves using RNeasy Plant Mini Kit (Qiagen, Courtaboeuf, France). RNA quality was determined with Nanodrop One/One^C^ (Thermo Fisher Scientific, USA) by analyzing their absorbance ratios A260/280 and A260/230, which were found to range between 2.0 and 2.2. Moreover, RNA quality was also examined with Bioanalyzer 2100 (Agilent, France) and a minimal RNA integrity number (RIN) of 8 was required for all samples. For microarray analyses, hybridization for all conditions were performed in triplicate with three sets of total RNA extracted from bulked wheat leaves (see above Sampling design section). Wheat Gene Expression Microarrays GE 4x44 (Agilent, Santa Clara, CA, USA) were used to study the gene expression profile between the different conditions. RNA amplification, staining, hybridization, and washing steps were conducted according to the manufacturer’s specifications. Slides were scanned at 5 µm/pixel resolution using the GenePix 4000B scanner (Molecular Devices Corporation, Sunnyvale, CA, USA). Images were used for grid alignment and expression data digitization with GenePix Pro 6.0 software (Molecular Devices Corporation, Sunnyvale, CA, USA). Gene expression data were normalized by Quantile algorithm. The three control samples were filtered for *P* < 0.05 and the average was calculated for each gene. A fold change (FC) value was calculated for each gene between individual treated samples and the mean of corresponding controls. Differentially expressed genes (DEGs) were selected for a significant threshold > 2.0 or < 0.5 (*P* < 0.05). Functional annotation of DEGs was based on NCBI GenBank and related-genes physiological processes were assigned with NCBI, AmiGO 2 Gene Ontology and UniProt. KEGG pathway analysis was also used to identify relevant biological pathways for the selected genes. All microarray data have been submitted to the NCBI GEO: archive for functional genomics data with the accession number GSE169298.

### 2.7 Metabolite extraction and UHPLC-MS analyses

Metabolites were extracted from powdered freeze-dried wheat leaves (30-50 mg per sample) using 25 µL of methanol per mg dry weight. The extract was then incubated in an ultrasound bath for 10 minutes, before centrifugation at 13000 g at 10°C for 10 minutes. The supernatant was analyzed using a Dionex Ultimate 3000 UHPLC system (Thermo Fisher Scientific, USA). The chromatographic separations were performed on a Nucleodur C18 HTec column (150 × 2 mm, 1.8 μm particle size; Macherey-Nagel, Germany) maintained at 30°C. The mobile phase consisted of acetonitrile/formic acid (0.1%, v/v, eluant A) and water/formic acid (0.1%, v/v, eluant B) at a flow rate of 0.3 mL.min^-1^. The gradient elution was programed as follows: 0 to 1 min, 95% B; 1 to 2 min, 95% to 85% B; 2 to 7 min, 85% to 0% B; 7 to 9 min, 100% A. The sample volume injected was 1 μL. The UHPLC system was coupled to an Exactive Orbitrap mass spectrometer (Thermo Fischer Scientific, USA), equipped with an electrospray ionization (ESI) source operating in positive mode. Parameters were set at 300°C for ion transfer capillary temperature and 2500 V for needle voltages. Nebulization with nitrogen sheath gas and auxiliary gas were maintained at 60 and 15 arbitrary units, respectively. The spectra were acquired within the m/z (mass-to-charge ratio) mass ranging from 100 to 1000 atomic mass units (a.m.u.), using a resolution of 50,000 at m/z 200 a.m.u. The system was calibrated internally using dibutyl-phthalate as lock mass at m/z 279.1591, giving a mass accuracy lower than 1 ppm. The instruments were controlled using the Xcalibur software (Thermo Fischer Scientific, USA). LC-MS grade methanol and acetonitrile were purchased from Roth Sochiel (France); water was provided by a Millipore water purification system. Apigenin and chloramphenicol (Sigma-Aldrich, France) were used as internal standards.

Metabolites belonging to different chemical families were identified based on published works about benzoxazinoids (de Bruijn et al., 2016), flavonoids (Wojakowska et al., 2013) and hydroxycinnamic acid amides (Li et al., 2018) from wheat. Putative metabolite identifications were proposed based on expertized analysis of the corresponding mass spectra and comparison with published literature. Further information was retrieved from the KEGG (Kyoto Encyclopedia of Genes and Genomes, http://www.genome.ad.jp/kegg/) and PubChem (http://pubchem.ncbi.nlm.nih.gov) databases. Relative quantification of the selected metabolites was performed using the Xcalibur software. For some metabolites, identity was confirmed with the corresponding standard provided by Sigma-Aldrich (France).

### 2.8 Statistical analyses

Protection efficacy data set was analyzed using One-Way analysis of variance (ANOVA) at *p* ≤ 0.05, while data obtained for *in planta* spore germination and hyphal growth were analyzed using ANOVA followed by the Tukey’s test at *p* ≤ 0.05, using GraphPad Prism software version 9 (GraphPad Software Inc., San Diego, USA). Regarding the *in vitro* antifungal activity assay, the half-maximal inhibitory concentration (IC_50_) was also determined with the GraphPad Prism software version 9. Differential metabolomic analyses were performed with the W4M platform (Guitton et al., 2017), using the Tukey’s Honest Significant Difference method followed by a false discovery rate (FDR) correction using the Benjamini-Hochberg procedure (Benjamini and Hochberg, 1995). Metabolites of interest were considered differentially accumulated when the false discovery rate was below 5% (FDR ≤ 0.05).

## 3 Results

### 3.1 Foliar application of mycosubtilin protects wheat against *Z. tritici* and reduces fungal spore germination and hyphal epiphytic growth

**Figure 1:**
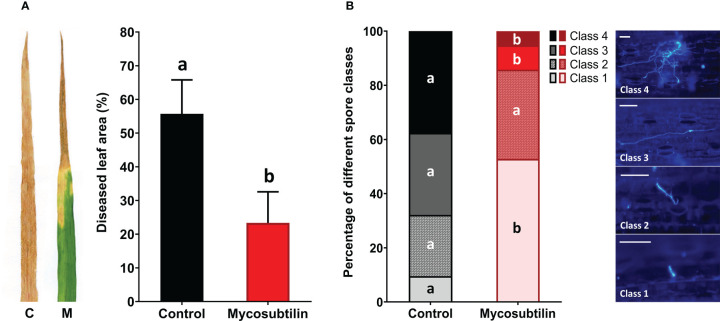
Disease severity level **(A)** and rates of *in planta* spore germination and epiphytic growth **(B)** of *Zymoseptoria tritici* (T02596 strain) on wheat leaves (cv. Alixan) pre-treated with mycosubtilin (M) at 100 mg.L^-1^ or not (C, control). **(A)** Disease severity level was scored 21 days after inoculation (*i.e* 23 days after mycosubtilin treatment) by assessing the area of lesions (chlorosis and necrosis) on the third leaf of each wheat plant (n=36). Means tagged with the same letter are not significantly different according to One-way ANOVA test (*P* ≤ 0.05). **(B)** Proportions of four different development stages of fungal spores were assessed by using Calcofluor staining recorded three days after inoculation (*i.e.* five days after mycosubtilin treatment). The different spore development classes are defined as followed: Class 1, non-germinated spore; Class 2, germinated spore with a small germ tube; Class 3, germinated spore with a developed germ tube; Class 4, germinated spore with a strongly developed germ tube. For each condition, the different classes were determined from 100 distinct spores chosen randomly on each third-leaf segment. Within each spore class, the presence of different letters indicates a significant difference according to the Tukey test at *p* ≤ 0.05. Scale bar = 25 µm.

The ability of mycosubtilin to protect wheat against *Z. tritici* was assessed in the greenhouse using the wheat cv. Alixan and the pathogenic *Z. tritici* strain T02596. At 21 days after inoculation, the disease severity level was 55.7% in the non-treated inoculated plants. Preventive foliar application of mycosubtilin at 100 mg.L^-1^ two days before inoculation resulted in significant disease reduction (23.3% of diseased leaf area, corresponding to a 58.1% decrease) in treated plants when compared to non-treated inoculated plants (**Figure 1A**). *In planta* fungal staining assays using Calcofluor revealed that the treatment with mycosubtilin significantly reduced the rates of both spore germination and epiphytic growth of *Z. tritici* at three days after inoculation, *i.e* five days after treatment (D5) (**Figure 1B**). In treated wheat plants, non-germinated spores (class 1) were significantly more abundant (approximatively five-fold) than in the control plants. Moreover, the number of germinated spores with either developed germ tube (class 3) or strongly developed germ tube (class 4) was significantly reduced (by three- and seven-fold, respectively) on treated plants when compared to the control ones.

### 3.2 Mycosubtilin impacts *in vitro* growth of both *Z. tritici* and wheat single cells, but at different concentration thresholds

**Figure 2:**
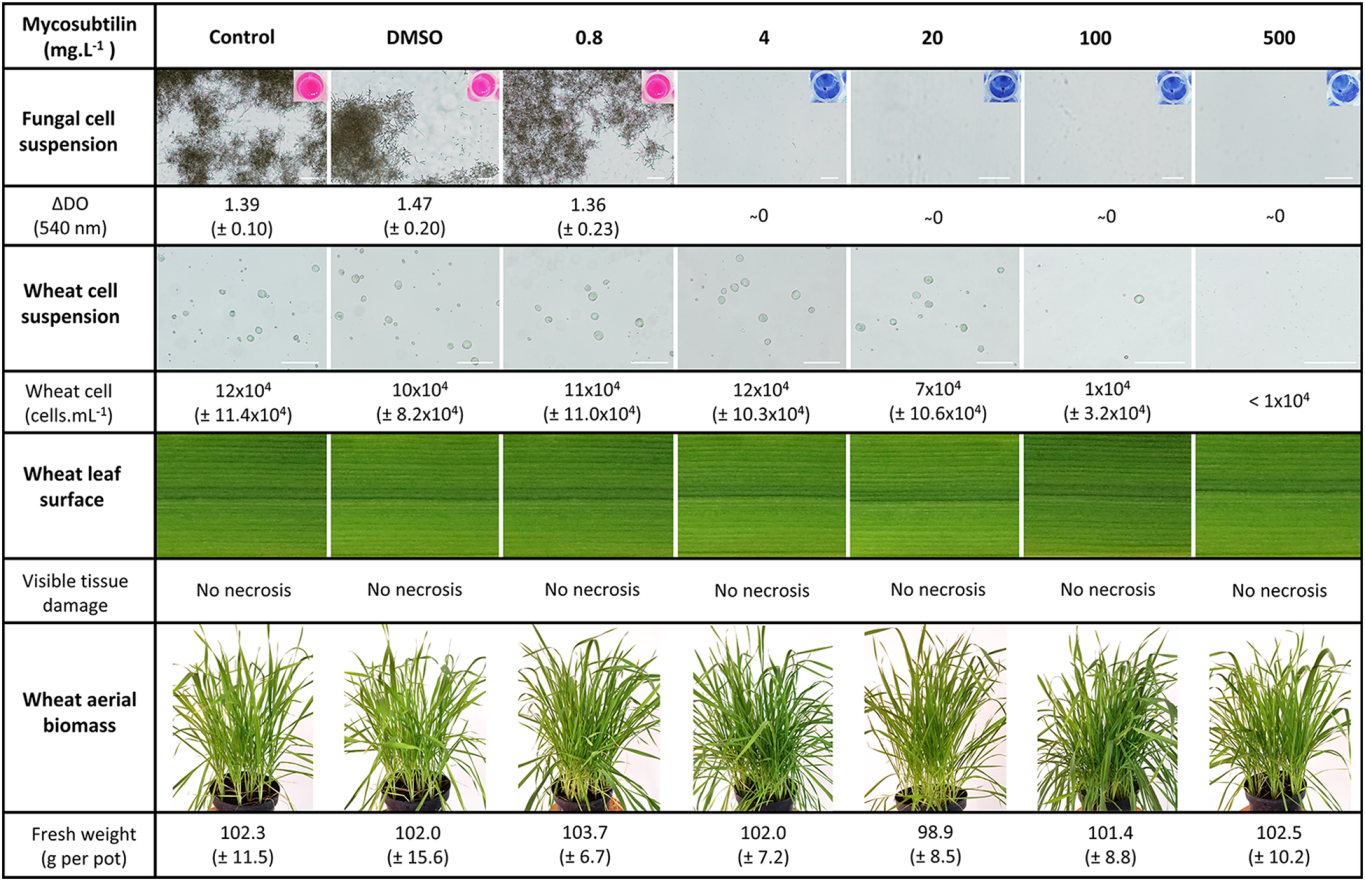
Effect of mycosubtilin at different concentrations on the growth of either *Zymoseptoria tritici* or wheat cells in liquid medium *in vitro* in twelve well microplates, on leaf appearance and on plant fresh biomass of whole plants grown in the greenhouse. Fungal growth was assessed using resazurin staining and optical density measurement 10 days after inoculation (dai) of glucose peptone medium with *Z. tritici* spores (n=6), while wheat cell growth was evaluated using Malassez hemocytometer at 21 days after inoculation of Gamborg B5 medium with wheat cell suspension (n=10). Leaf appearance was assessed visually at two, seven and fifteen days after plant treatment (n=27), while the fresh biomass was determined by weighting nine whole plants from each pot (n=3). Representative pictures of either *Z. tritici* or wheat cell cultures observed under a light microscope at magnification 10x and 20x respectively, as well as the microplate wells used for *Z. tritici* culture medium staining with resazurin, are shown. Likewise, representative pictures of leaf appearance and plant fresh aerial biomass, at fifteen days after treatment, are also presented. Scale bar = 100 µm.

The effect of mycosubtilin on the *Z. tritici in vitro* growth was evaluated on solid PDA medium in 12-well plates. The biomolecule exhibited a strong antifungal activity against the pathogen, with IC_50_ and MIC values of 0.57 and 0.78 mg.L^-1^, respectively (**Supplementary Figure S2**). In order to gain more insights into the mode of action of mycosubtilin on the host plant as well as on the pathogen, further bioassays were performed in liquid medium to examine the effect of the lipopeptide on the development (cell multiplication) of either wheat single cells or *Z. tritici* spores. Microscopic observation of the fungal spores grown in glucose peptone liquid medium showed that mycosubtilin totally inhibits the fungal growth from the concentration of 4 mg.L^-1^. Besides, the lipopeptide exhibits also an effect on the growth of wheat single cells cultivated in suspension in MS liquid medium, but with a total inhibiting concentration of 500 mg.L^-1^ (**Figure 2**). Hence, the activity threshold of mycosubtilin seems to be 125-fold higher towards wheat single cells than against *Z. tritici* spores in liquid medium. However, no phytotoxic effect of mycosubtilin was observed at this concentration when the biomolecule is applied on wheat leaves. Indeed, no visible leaf necrosis and no significant effect on the total fresh biomass were observed on the treated plants at all tested concentrations (**Figure 2**).

### 3.3 Mycosubtilin regulates several defense-associated genes in wheat against *Z. tritici*

Transcriptomic analyses using RNA microarray assay were performed in order to examine both eliciting and priming effects of mycosubtilin in wheat towards *Z. tritici* during the early stages of the fungal infection. The bioassay was performed in non-inoculated conditions at two days after treatment (D2), and in both non-inoculated and inoculated conditions at five days after treatment (D5), *i.e* three days after inoculation. The eliciting effect was examined at D2 and D5 by comparing the treated non-inoculated plants to non-treated and non-inoculated plants. The priming effect was investigated at D5 by comparing treated and inoculated plants to non-treated inoculated plants, and by taking into account the elicitation modality at both D2 and D5. Moreover, in order to have a reliable conclusion and comprehensive view on the data linked to the priming effect, additional comparisons (treated and inoculated plants *versus* treated non-inoculated plants, and treated and inoculated plants *versus* non-treated non-inoculated plants), were also performed (**Supplementary Table S1** and **Figures S3**, **S4**). The fungal effect was investigated at D5 by comparing non-treated and inoculated plants to non-treated and non-inoculated ones. The different comparisons will thereafter be referred to as eliciting, fungal, and priming effects, corresponding to eliciting, infection alone, and priming modalities, respectively.

**Figure 3:**
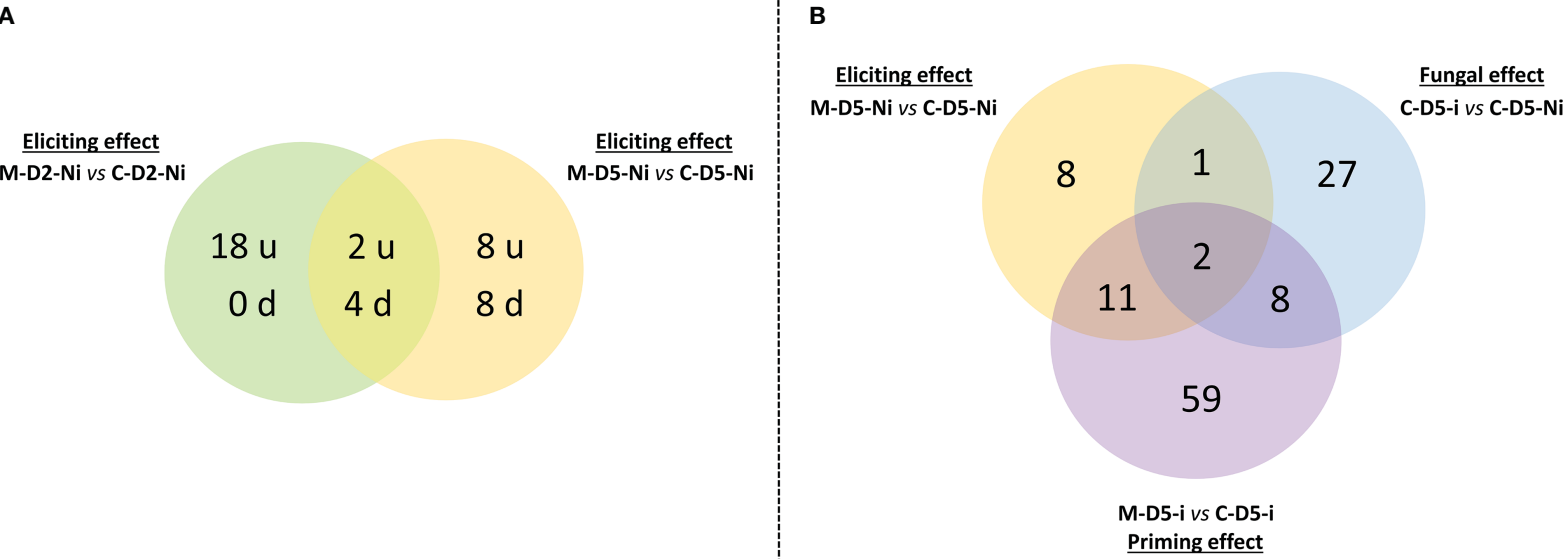
Venn diagrams underlying **(A)** the number of up-regulated (u) or down-regulated (d) genes observed after mycosubtilin treatment in non-inoculated conditions with *Zymoseptoria tritici* as well as **(B)** the number of differentially expressed genes in the different tested conditions at five days after treatment with mycosubtilin (*i.e.* three days after wheat inoculation with *Z. tritici*). In **(A)**, the potential early elicitation effect of mycosubtilin at two days after treatment (M-D2-Ni vs C-D2-Ni) on the levels of wheat leaf gene expression is compared to the later one at five days after treatment (M-D5-Ni vs C-D5-Ni). In **(B)**, The eliciting effect of mycosubtilin (M-D5-Ni vs C-D5-Ni) is compared to the fungus effect (C-D5-i vs C-D5-Ni) and to the priming effect of mycosubtilin (M-D5-i vs C-D5-i). M refers to plants treated with mycosubtilin whereas C stands for mock treated ones. Ni stands for mock inoculated whereas i indicates that plants were inoculated with *Z. tritici*. D2 and D5 signify that leaves were sampled respectively at two and five days after mycosubtilin treatment.

**Figure 4:**
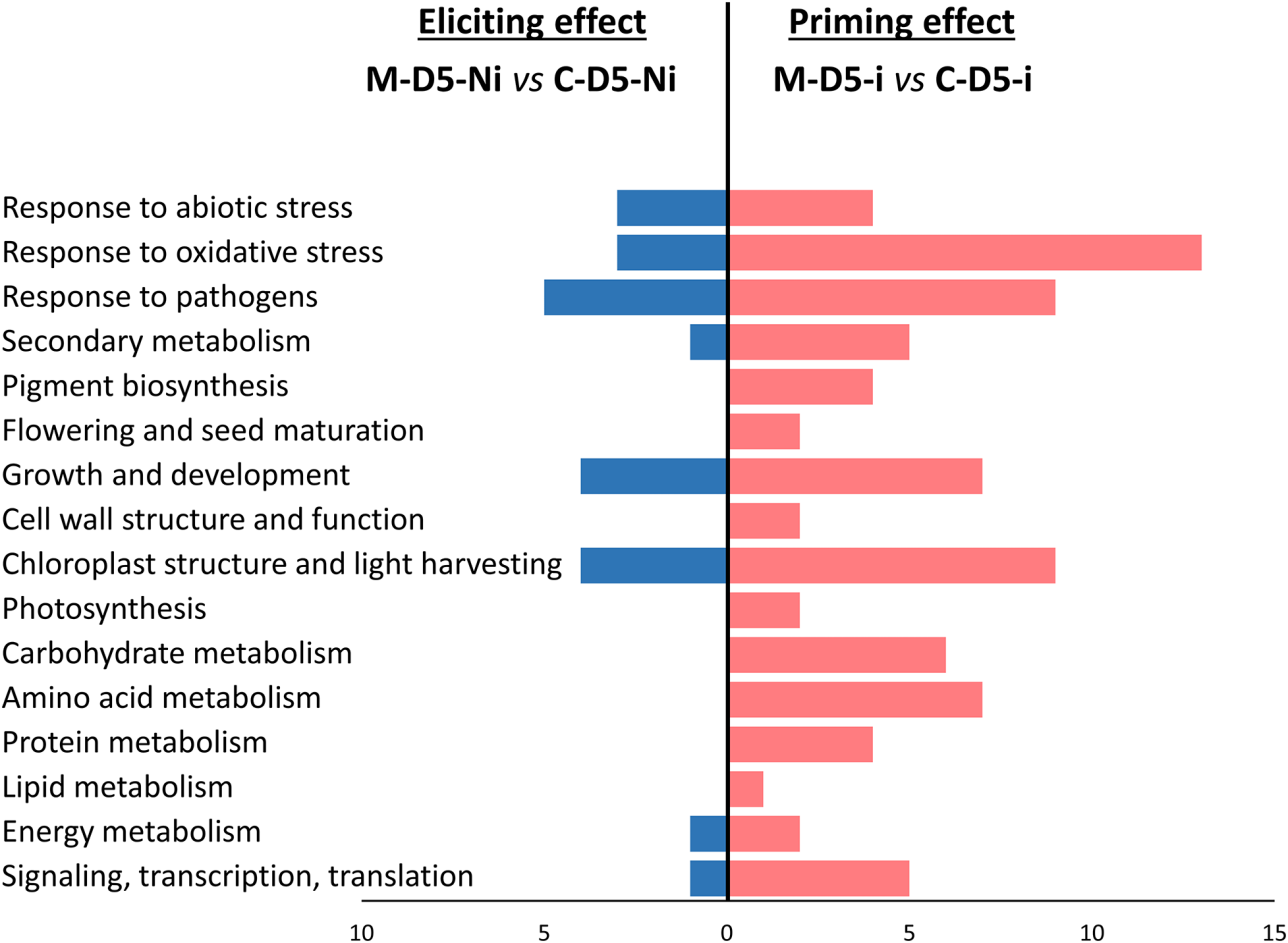
Functional groups of differentially expressed genes (DEGs) highlighting eliciting (M-D5-Ni vs C-D5-Ni) or priming (M-D-5i vs C-D5-i) effects of wheat defenses after treatment with mycosubtilin. Functional annotation of DEGs was performed based on NCBI GenBank and related-gene physiological processes were assigned with NCBI, AmiGO 2 Gene Ontology and UniProt. M refers to plants treated with mycosubtilin whereas C stands for mock treated ones. Ni stands for mock inoculated whereas i indicates that plants were inoculated with *Zymoseptoria tritici*. D5 signifies that leaves were sampled at five days after mycosubtilin treatment.

**Figure 5:**
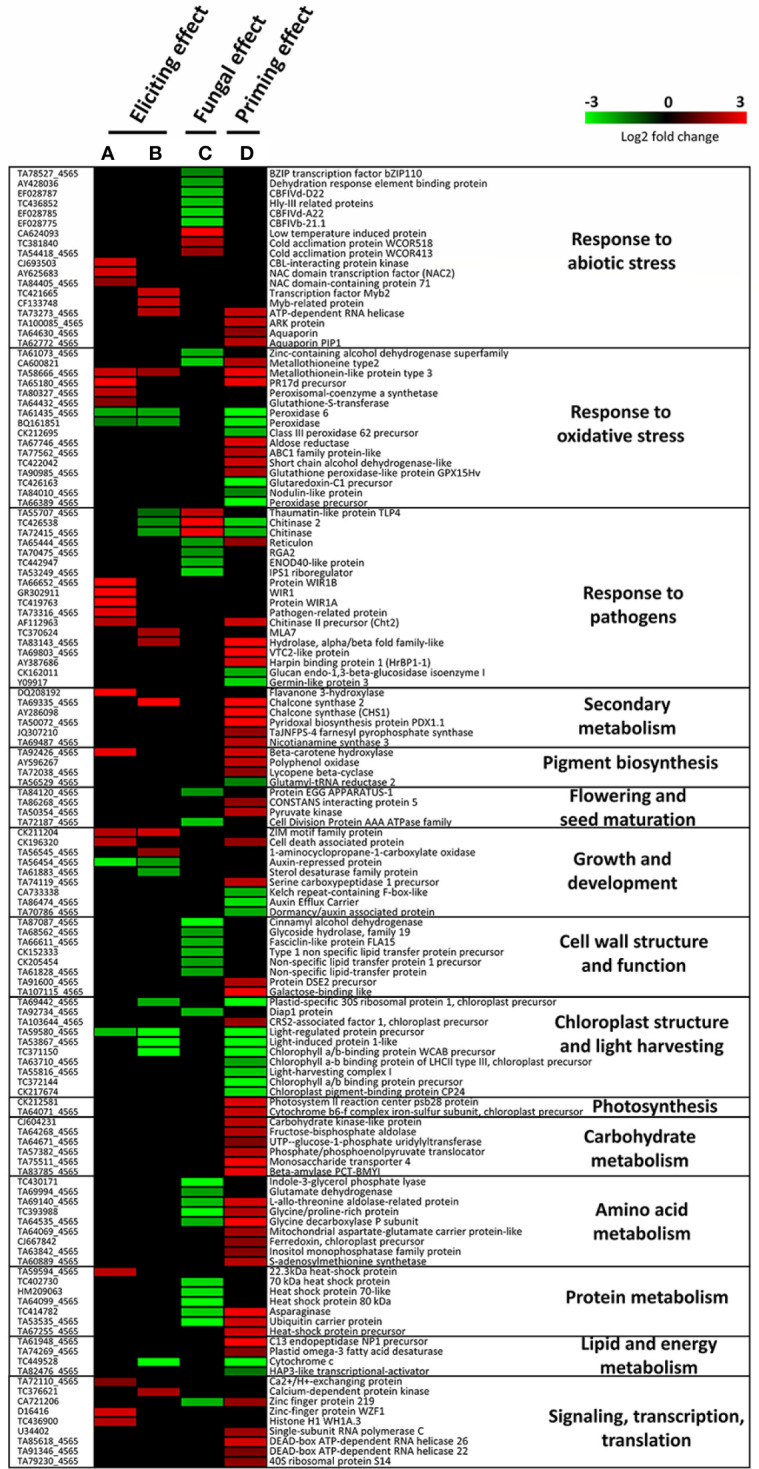
Heatmap showing significantly up- or down-regulated genes in wheat third-leaves (cv. Alixan) treated or not with 100 mg.L^-1^ mycosubtilin and inoculated or not with *Zymoseptoria tritici* (strain T02596), recorded at two days after treatment in non-inoculated conditions **(A, B)** and at five days after treatment (*i.e.* three days after inoculation) in inoculated conditions **(C, D)**. A and B highlight the eliciting effect, C shows the fungal effect, while D reveals the priming effect induced by mycosubtilin. Gene-related physiological processes are represented on the right part of the graph and were determined using NCBI, AmiGO 2 Gene Ontology, KEGG and UniProt. **(A)**, M-D2-Ni *vs* C-D2-Ni; **(B)**, M-D5-Ni *vs* C-D5-Ni; **(C)**, C-D5-i *vs* C-D5-Ni; **(D)**, M-D5-i *vs* C-D5-i. Significant relative change in gene expression is presented in Log2 ratio, according to the color scale, using the WebMev software. M refers to plants treated with mycosubtilin whereas C stands for mock treated ones. Ni stands for mock inoculated whereas i indicates that plants were inoculated with *Z. tritici*. D2 and D5 signify that leaves were sampled respectively at two and five days after mycosubtilin treatment.

A total of 130 genes were regulated (*i.e.* when taking into account both up and down regulations) when examining eliciting, fungal, and priming effects and considering all time points (**Supplementary Table S2**). Overall, treatment with mycosubtilin led to a broader gene regulation response in priming modality at D5 when compared to both elicitation modalities at D2 or D5 (**Figures 3**-**5**). When considering the eliciting effect at both D2 and D5, only 40 differentially expressed genes (DEGs) were highlighted, with 18, 6, and 16 DEGs specifically scored at D2, both D2 and D5, and D5, respectively. Among them, 28 were upregulated and 12 were downregulated (**Figure 3A**). At D5, priming modality displayed considerably more DEGs (80) when compared to the eliciting effect at D5 (22), with 13 DEGs found in common among the two modalities. Globally, when considering all investigated modalities at D5 (eliciting, fungal, and priming effects), a total of 116 DEGs were recorded (**Figure 3B**). Out of the detected 116 DEGs, 8, 27 and 59, were specifically noticed during either elicitation, fungal or priming effect modalities, respectively. (**Figure 3B**).

Functional groups of DEGs recorded during wheat eliciting or priming by mycosubtilin at D5 were compared (**Figure 4**). Consistently with the findings highlighted above in Venn diagrams, priming conditions clearly exhibited more DEGs annotated in the highlighted functional groups when compared to the eliciting conditions at D5. Among the sixteen identified functional groups of DEGs, those linked to stress responses were the most regulated upon treatment with mycosubtilin, especially in priming conditions, followed by those involved in chloroplast and light harvesting, as well as growth and development. Few functional groups of DEGs were found only in priming modality, including those related to pigment biosynthesis, photosynthesis, flowering and seed maturation, cell wall structure and function, and primary metabolic pathways (carbohydrate, amino acid, protein, and lipid metabolisms). Moreover, a relative strong increase in the number of DEGs involved in transcription regulation, RNA-processing and translation, as well as secondary metabolism, was observed in priming when compared to elicitation modalities (**Figure 4**).

When examining the gene regulation in detail, mycosubtilin-induced eliciting effect observed at D2 mainly involved DEGs associated to responses to pathogens, oxidative stress, and abiotic stress; most of them were significantly upregulated, except *peroxidase* and *peroxidase 2*, which were significantly downregulated (**Figure 5** and **Supplementary Table S2**). Noticeably, three DEGs involved in signaling, transcription, and translation (*Ca^2+^/H^+^ exchanging protein*, *zinc finger protein WZF1*, and *histone H1 WH1A.3*) were found overexpressed at D2 (**Figure 5**). Additionally, at D5, a downregulation of eight other DEGs, three associated with responses to pathogens (*thaumatin-like protein TL4, chitinase*, and *chitinase*2), as well as an upregulation of eight DEGs, with two linked with responses to pathogens, were recorded. In the priming modality at D5, mycosubtilin induced the regulation of a substantial number of genes related to several physiological pathways. Among them, a subset of 26 genes is linked to responses to stresses, including thirteen to oxidative stress, nine to pathogens, and four to abiotic stress. Interestingly, several genes involved in abscisic acid (ABA) biosynthesis (*beta-carotene hydroxylase* and *lycopene β-cyclase*) and ABA-associated signaling pathways (*ARK protein*, *aquaporins*, *aldose reductase*, *ABC1 family protein-like*, *glutathione peroxidase-like protein GPX15Hv*, *nicotianamine synthase 3*, *chlorophyll a/b binding protein of LHCII type III chloroplast precursor*, *light-harvesting complex 1* and *S-adenosylmethionine synthetase*) were also regulated in priming modality at D5 (**Figure 5**). Regarding the effect of the fungal infection alone on the wheat leaf transcriptome at D5, only an upregulation of six DEGs involved in response to abiotic stress and pathogens was obtained, whereas a set of 32 DEGs was downregulated by the fungus alone at this time point, among them some are also related to responses to abiotic stress and pathogens. However, the most remarkable effect of *Z. tritici* is the downregulation of an important number of genes involved in cell-wall structure, amino acid metabolism, and protein metabolism (**Figure 5**).

### 3.4 Mycosubtilin primes flavonoid accumulation in wheat against *Z. tritici*

Metabolomic analyses using UHPLC-MS were undertaken to assess the effect of mycosubtilin on the wheat leaf metabolome in both non-inoculated and inoculated conditions, and at the same time points than those targeted in the above described transcriptomic assay (D2 and D5), with an additional time point corresponding to 15 days after treatment (D15), *i.e* 13 days after inoculation, corresponding to the late stage of *Z. tritici* biotrophic phase. The eliciting effect was investigated at D2, D5, and D15 by comparing the treated non-inoculated plants to non-treated and non-inoculated plants. The priming effect was examined at D5 as well as D15, on the one hand, by comparing treated and inoculated plants to non-treated inoculated plants, and on the other hand, by considering the elicitation modalities at D2, D5, and D15. Besides, further comparisons (treated and inoculated plants *versus* treated non-inoculated plants, and treated and inoculated plants *versus* non-treated non-inoculated plants), were also carried out in order to have a comprehensive picture on the data linked to the priming effect on leaf metabolome (**Supplementary Figure S5**). As described in the transcriptomic assay, the different comparisons in the metabolomic assay will also be referred to as eliciting, fungal, and priming effects, corresponding to eliciting, infection alone, and priming modalities, respectively.

**Figure 6:**
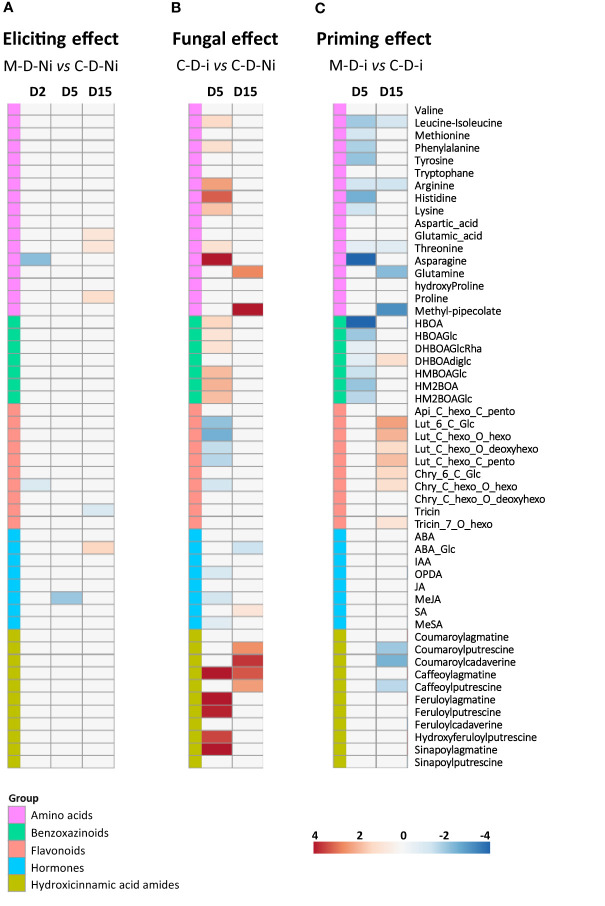
Heatmap of significant relative changes in metabolite patterns within wheat third-leaves (cv. Alixan) treated or not with 100 mg.L^-1^ mycosubtilin and inoculated or not with *Zymoseptoria tritici* (strain T02596) at different time points (n = 9 plants). **(A)**, potential elicitation effect of mycosubtilin in non-inoculated conditions at 2 (M-D2-Ni vs C-D2-Ni), 5 (M-D5-Ni vs C-D5-Ni) and 15 (M-D15-Ni vs C-D15-Ni) days after treatment (dat). **(B)**, Fungal effect on wheat leaf metabolome at 5 (C-D5-i vs C-D5-Ni) and 15 (C-D15-i vs C-D15-Ni) dat, *i.e.* at 3 and 13 days after wheat inoculation (dai) with *Z. tritici*, respectively. **(C)**, Priming effect of mycosubtilin in inoculated conditions at 5 (M-D5-i vs C-D5-i) and 15 (M-D15-i vs C-D15-i) dat (*i.e.* at 3 and 13 dai, respectively). M refers to plants treated with mycosubtilin whereas C stands for mock treated ones. Ni stands for mock inoculated whereas i indicates that plants were inoculated with *Z. tritici*. D2, D5 and D15 signify that leaves were sampled respectively at two, five and fifteen days after mycosubtilin treatment. Log2 of significant metabolite fold changes for indicated pairwise comparisons are given by shades of red or blue colors according to the scale bar. Metabolites were grouped according to their functional or chemical family as amino acids, benzoxazinoids, flavonoids, hormones and hydroxycinnamic acid amides. Data represent mean values of nine biological replicates for each condition and time point. Statistical analyses were performed using the Tukey’s Honest Significant Difference method followed by a false discovery rate (FDR) correction, with FDR < 0.05. For FDR ≥ 0.05, Log2 fold changes were set to 0.

The analyses were targeted on 54 metabolites belonging to major chemical families that were detected and quantified using UHPLC-MS (**Figure 6** and **Supplementary Table S3**). Plant treatment with mycosubtilin and/or inoculation with *Z. tritici* resulted in marked differentiations among wheat leaf metabolite patterns, as highlighted by the PCA analysis (**Supplementary Figure S6**). The largest changes in the accumulation of the selected metabolites were observed during fungal infection alone or during priming modalities, whereas only minor modifications were scored in eliciting modalities (**Figure 6**). In all eliciting modalities, only eight differentially accumulated metabolites (DAMs) were recorded. Among them, four DAMs were under-accumulated, two at D2 (asparagine and chry-C-hexo-O-hexo), one at D5 (MeJA), and one at D15 (tricin), and four were over-accumulated at D15, including three amino acids (glutamic acid, threonine and proline) and one hormone (ABA-Glc). However, in the priming modalities, a higher number of DAMs was scored, including 16 DAMs at D5 and 16 DAMs at D15 (**Figure 6**). At D5, only metabolite down-accumulation was detected (corresponding mainly to those over-accumulated in the fungal modalities), whereas at D15, both increases and decreases in metabolite concentration were observed. At D15, a decrease in the concentration of three hydroxycinnamic acid amides (coumaroylputrescine, coumaroylcadaverine, and caffeoylputrescine) and five amino acids and derivatives (leucine-isoleucine, arginine, threonine, glutamine, and methyl-pipecolate) was found. Interestingly, seven metabolites belonging to flavonoids and one benzoxazinoid were over-accumulated specifically in the priming modality at D15 (**Figure 6**).

**Figure 7:**
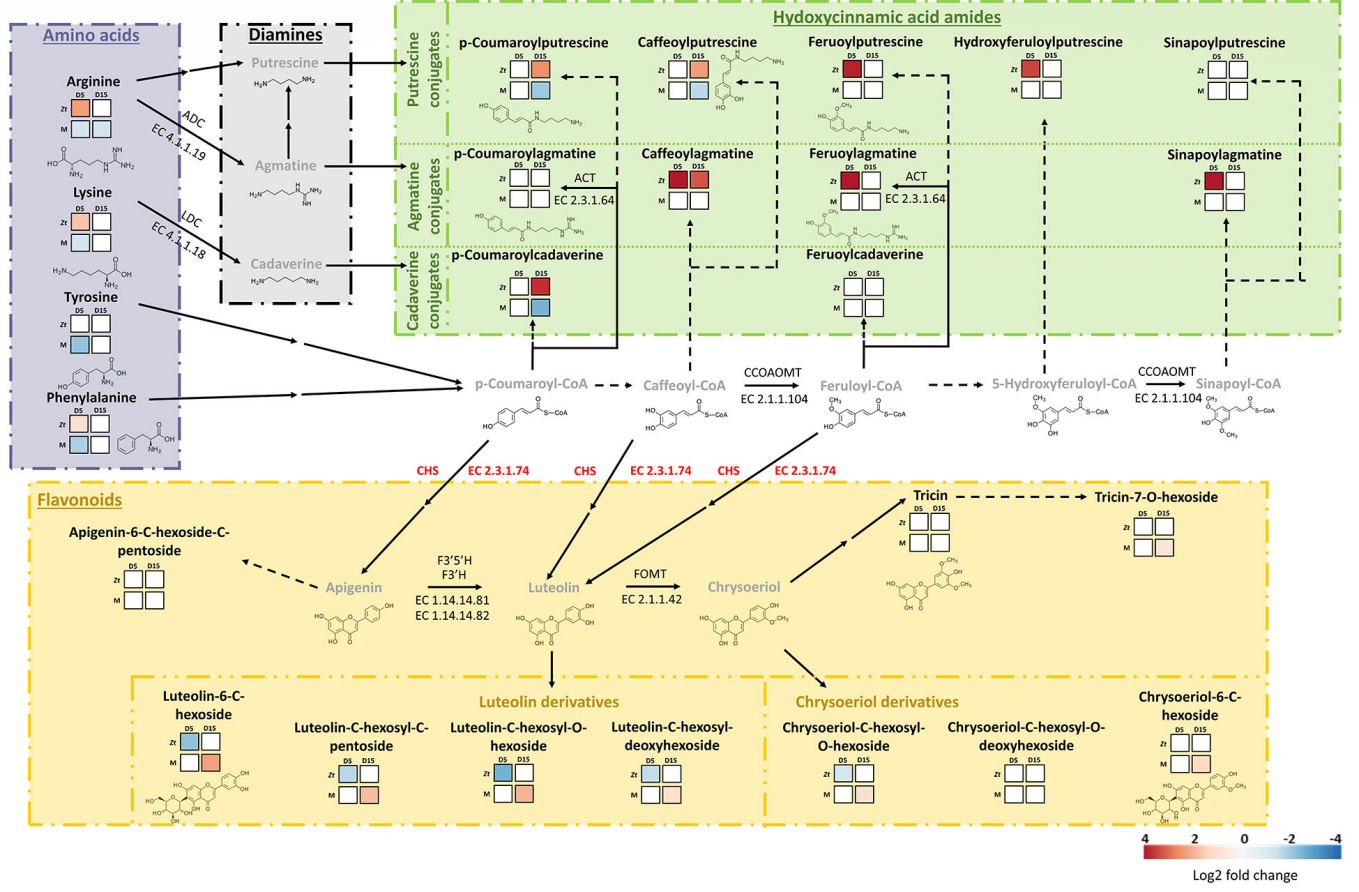
Metabolic map of the biosynthesis pathways and patterns of accumulation of major hydroxycinnamic acid amides and flavonoids within wheat third leaves (cv. Alixan) inoculated with *Zymoseptoria tritici* (strain T02596) pre-treated or not with mycosubtilin. Metabolic pathways are based on KEGG pathways (map00310, map00330, map00940, map00941, map00944). Amino acids, diamines, hydroxycinnamic acid amides and flavonoids metabolite families are separated and indicated in block of different colors, respectively purple, black, green and orange. Heatmaps show significant Log2 fold changes in metabolite accumulation, according to the corresponding scale bar. For each analyzed metabolite, the two upper squares represent the fungus effect on the relative accumulation of the compound, from the left to the right, at five (C-D5-i vs C-D5-Ni) and fifteen (C-D15-i vs C-D15-Ni) days after treatment (dat), *i.e* 13 days after inoculation. The two lower squares represent the effect of application of mycosubtilin (priming) prior to inoculation with *Z. tritici* on the wheat metabolome at five (M-D5-i vs C-D5-i) and fifteen (M-D15-i vs C-D15-i) dat. Metabolites in grey were not analyzed in this study. Solid arrows stand for enzymatic reactions completely described in the abovementioned KEGG pathways whereas dashed arrows are for suggested ones. Double arrows represent two or more metabolic steps. With ACT, agmatine N4-coumaroyltransferase; ADC, arginine decarboxylase; CCOAOMT, caffeoyl-CoA O-methyltransferase; CHS, chalcone synthase; F3’5’H, flavonoid 3’;5’-hydroxylase, F3’H, flavonoid 3’-monooxygenase; FOMT, flavone 3’-O-methyltransferase; LDC, lysine decarboxylase. Genes coding for chalcone synthase were significantly upregulated when plants were primed with mycosubtilin at five days after treatment (M-D5-i vs C-D5-i), hence CHS is presented in red. M refers to plants treated with mycosubtilin whereas C stands for mock treated ones. Ni stands for mock inoculated whereas i indicates that plants were inoculated with *Z. tritici*. D5 and D15 signify that leaves were sampled respectively at five and fifteen days after mycosubtilin treatment.

Fungal infection alone induced substantial modifications in metabolite accumulation patterns at D5 and D15, with marked changes at D5, corresponding to the early stages of infection (**Figure 6**). Indeed, at D15, only significant changes in the concentration of glutamine, methyl-pipecolate, salicylic acid, and four hydroxycinnamic acid amides were noticed. However, at D5, a total of 26 DAMs, about half of the quantified molecules, were significantly regulated, with 18 molecules over-accumulated and eight under-accumulated. Seven amino acids, six benzoxazinoids, and five hydroxycinnamic acid amides were more concentrated, while three phytohormone precursors or derivatives (OPDA, MeJA and MeSA), five flavonoids, four luteolin derivatives, and one chrysoeriol derivative were down-accumulated (**Figure 6**). The proposed model on the effects of mycosubtilin-induced priming and *Z. tritici* infection at D5 and D15 on both hydroxycinnamic acid amide and flavonoid biosynthesis pathways within wheat leaves is illustrated (**Figure 7**).

## 4 Discussion

We have combined here transcriptomic and metabolomic approaches to unravel the resistance mechanisms induced in wheat, using the *B. subtilis* lipopeptide mycosubtilin as a treatment and *Z. tritici* as a phytopathogenic model. Only few previous works have used Omics to characterize the induced resistance in plants, whereas such tools could be helpful and informative about the molecular and physiological changes occurring during plant resistance activation using exogenous treatments (*e.g.* Gauthier et al., 2014; Fiorilli et al., 2018). Our results revealed that mycosubtilin likely acts on the wheat-*Z. tritici* pathosystem through a double activity (direct and indirect) and that the indirect activity relies mainly on the priming of wheat defense mechanisms.

### 4.1 Mycosubtilin displays direct antifungal activity towards *Z. tritici* and likely interacts with wheat leaf cells without causing *in planta* phytotoxicity

Foliar application of mycosubtilin at 100 mg.L^-1^ decreased *Z. tritici* symptoms on wheat plants by more than half (58.1%), thus agreeing with previous results obtained on this pathosystem (Mejri et al., 2018). Mycosubtilin has also been reported to biocontrol other pathogens, such as *Bremia lactucae* on lettuce, *Botrytis cinerea* on grapevine, and *Fusarium oxysporum* f. sp. *iridacearum* on *Iris* sp. (Deravel et al., 2014; Farace et al., 2015; Mihalache et al., 2018). Our *in vitro* and *in planta* bioassays have shown that mycosubtilin exhibits a direct antifungal effect on both spore germination and epiphytic growth of *Z. tritici* on wheat leaves, hence corroborating previous findings on this fungus (Mejri et al., 2018). Interestingly, our results revealed that mycosubtilin exhibits growth inhibiting activity on fungal as well as wheat single cells grown in suspension, suggesting that the lipopeptide interacts with the cells of both organisms with a common process. Indeed, it has been reported that the mode of action of mycosubtilin could rely on the destabilization of cytoplasmic membranes (Nasir et al., 2010; Nasir and Besson, 2012), that could lead to loss of cell integrity, cytoplasm leakage, and ultimately cell death. We can, thus, hypothesize that the biological activities of mycosubtilin on both *Z. tritici* and wheat cells may be associated with its ability to interact with the plasma membranes of both organisms. Nevertheless, the *in vitro* threshold concentration of the molecule activity on both species varies strongly, with a difference of 125-fold between *Z. tritici* and wheat cells, even though fungal cells growing in contact with the biomolecule during the bioassay were protected by cell-walls, while wheat cells grown in suspension didn’t likely develop complete cell-walls. However, no visible phytotoxicity caused by mycosubtilin on the whole plants was observed even at the highest tested concentration (500 mg.L^-1^), presumably because leaves are overall more robust (presence of cuticle, etc.) than single wheat cells growing *in vitro*. An effect of mycosubtilin at 50 mg.L^-1^ on the growth of grapevine single cells has previously been observed, with a significant increase in the amounts of cell death (23% of the total cells compared with 11% in the control) observed at 24h post-treatment, thus agreeing with our results (Farace et al., 2015). Taken together, these results suggest that, when applied on wheat plants at 100 mg.L^-1^, mycosubtilin is able, on the one hand, to display direct antimicrobial activity towards the pathogen and, on the other hand, to interact with the plasma membranes of plant leaf cells without causing damages that could lead to their death. More likely, this interaction of the molecule with the plasma membranes of plant cells could be the initial stimulus triggering the downstream defense reactions highlighted in wheat plants in the transcriptomic and metabolomic assays. This defense-initiating stimulus could be attributed, as suggested in other plant models, to the potential biophysical and biochemical interactions of mycosubtilin with the lipid bilayer of the leaf cell plasma membranes (Crouzet et al., 2020). The insertion of the lipopeptide within them could, hence, modify their fluidity as an “abiotic stress-like” agent, like a thermal or drought stress (Niu and Xiang, 2018; Couchoud et al., 2019). This “abiotic stress-like” mechanism could therefore explain the accumulation of gene transcripts involved in plant defenses against abiotic and oxidative stresses found in eliciting conditions.

### 4.2 Perception of mycosubtilin by wheat leaves leads to the elicitation of few genes associated with plant defense

Transcriptomic analyses revealed that in wheat plants treated with mycosubtilin (100 mg.L^-1^), the expression of genes involved in plant responses to stresses and other biological functions was significantly regulated. At D2, in the elicitation modality, 24 DEGs were recorded, among which, three involved in signaling, transcription and translation were upregulated, including a gene encoding for Ca^2+^/H^+^ exchanging protein, a crucial regulatory protein of cytoplasm calcium homeostasis. Moreover, we found in the same modality and time point an overexpression of a *CBL-interacting protein kinase* (*CIPK*) gene, encoding for a protein functioning as a calcium sensor displaying a large range of activity in plant responses to stresses (Cui et al., 2018). Calcium influx into the cytoplasm is considered as one of the first cellular reactions after stress signal perception, that can trigger subsequent downstream defense reactions upon detection of the elevation of calcium concentration, by calcium‐dependent protein kinases (CDPKs) (Harmon et al., 2000). The accumulation of *Ca2+/H+ exchanging protein* and *CIPK* transcripts detected in our conditions could likely be linked to the mycosubtilin-induced initial stimulus suggested above on the wheat cell plasma membranes, leading to a modification in Ca^2+^ concentration inside wheat cells and hence to the subsequent triggered defense reactions observed in wheat leaves. In addition, among the DEGs still highlighted at D2 in the elicitation modality, 14 are associated to responses to either oxidative, pathogen, or abiotic stresses, suggesting that mycosubtilin confers to wheat an increased resistance level to these stresses. Notably, the upregulation of three genes encoding for a Pathogenesis-related (PR) protein or precursors (*pathogen-related protein*, c*hitinase II precursor*, and *PR17d precursor*), known to play a major role in plant resistance to pathogens, was observed. Chitinases are enzymes known to be involved in wheat defenses against *Z. tritici* by degrading the chitin of fungal cell-wall (Kettles and Kanyuka, 2016). PR-17 is a class of PR proteins with an unknown mode of action, discovered by Christensen et al. (2002) and which could be involved in wheat defense against powdery mildew (Görlach et al., 1996). WIR1 is a membrane protein that was reported to contribute to wheat defense responses against fungal infections, including powdery mildew and stripe rust, supposedly by increasing the adhesion of the cellular membrane to the cell wall during the pathogen attack (Bull et al., 1992; Coram et al., 2008). The accumulation of these transcripts at D2, *i.e* just before the moment of plant inoculation, may contribute to wheat protection against *Z. tritici*. Nevertheless, further investigations are needed to determine the role of these proteins during the studied compatible interaction.

At D5, still in the elicitation context, 22 DEGs were found, six of them were also reported at D2, including three involved in responses to oxidative stress (*peroxidase*, *peroxidase 6* and *metallothionein-like protein type 3*) and two in growth and development (*ZIM motif family protein* and *auxin-repressed protein*). Genes encoding for ZIM motif family protein have been described as key repressors of the jasmonic acid (JA) signaling pathway (Chini et al., 2009), being, hence, involved in the regulation of many physiological processes, especially in the mediation of plant responses to biotic and abiotic stresses (Sun et al., 2017; Ruan et al., 2019). A large number of genes belonging to this family have been found to be regulated by other hormones such as gibberellins and ABA (Zheng et al., 2020). Considering DEGs involved in the growth and development functional group, the upregulation of 1*-aminocyclopropane-1-carboxylate (ACC) oxidase* is to be noticed. This enzyme has been shown to be of prior importance for ethylene (ETH) production in plants (Houben and Van de Poel, 2019). As ETH is a gaseous plant hormone playing a crucial role in several biological processes, including tolerance to stresses, wheat responses to mycosubtilin could hence include ETH-responses (Adie et al., 2007a). Remarkably, Chandler et al. (2015) showed that mycosubtilin induces changes in gene expression in rice cells *in vitro*, with, especially, an increase in the amount of *ACC synthase* (*ACS1*) transcripts, another key enzyme in ETH synthesis. Regarding DEGs involved in response to pathogens, genes encoding for PR proteins (*thaumatin-like protein TLP4* and *chitinases*) were found to be downregulated, whereas *MLA7*, an homolog of a resistance gene against powdery mildew in barley (Caldo et al., 2004), and *hydrolase alpha/beta fold family-like*, encoding for a protein family displaying large varieties of functions in plants (Mindrebo et al., 2016), were upregulated. In addition to this ability of mycosubtilin to regulate wheat defenses against biotic stress, we also observed significant modifications in the expression of genes involved in responses to abiotic stress, suggesting that mycosubtilin triggers multifaceted cross-talks in wheat defense pathways. In fact, three DEGs involved in response to abiotic stress were upregulated in the elicitation modality at D5, including genes encoding for transcription factor (TF) MYB2 (myeloblastosis), MYB-related protein, and ATP-dependent RNA helicase. MYB TFs were particularly studied for their critical importance in plant growth and stress tolerance, as reviewed by Ambawat et al. (2013). More precisely, a MYB2 was reported to participate in rice tolerance to salt, cold, and dehydration stress (Yang et al., 2012). The authors also showed that this TF was regulated by ABA. ATP-dependent helicases are major actors involved in various biological processes involving RNA, such as RNA splicing and decay as well as translation initiation (Nakamura et al., 2004). Zhang et al. (2014) reported that wheat RNA helicase 1 (*TaRH1*) may play a regulatory role during plant responses to both biotic and abiotic stresses.

### 4.3 Mycosubtilin primes genes associated with several metabolic pathways in wheat

In the priming modality at D5, we observed DEG patterns different from those detected in the elicitation ones at D2 and D5, with a number of DEGs drastically higher than in the other tested modalities, suggesting that mycosubtilin acts on wheat as a priming compound. This hypothesis is supported by the upregulation of the gene encoding for histone H1 WH1A.3 recorded at D2 in elicitation modality. Indeed, histone modifications are supposed to play a central role in priming memorization and transcription reprogramming after the stressing cue, corresponding here to mycosubtilin application (Lämke and Bäurle, 2017). Out of the 80 DEGs noticed in the priming modality, 26 are involved in stress responses (oxidative, pathogen and abiotic stresses). Among them, we observed significant downregulation of the expression of genes encoding for chitinases, glucan endo-1,3- beta glucosidase isoenzyme 1 and a germin-like protein. In addition, as observed in elicitation modalities, transcripts of *peroxidase* were also downregulated, suggesting that mycosubtilin may negatively regulate peroxidase-related defense mechanisms. On the other hand, mycosubtilin priming led to the overexpression of DEGs involved in responses to pathogens such as *harpin binding protein (HrBP1-1)*, a gene encoding a protein located in plant cell walls known to promote anti-pathogen responses and SAR in plants (Chen et al., 2012). We also found DEGs potentially involved in carotenoids and ABA biosynthesis, such as *beta-carotene hydroxylase* and *lycopene β-cyclase* (Finkelstein, 2013). Remarkably, a significant number of DEGs described to play a part in ABA signaling and response pathways were regulated, such as genes encoding for ARK protein, aquaporins, aldose reductase, glutathione peroxidase-like protein GPX15Hv, nicotianamine synthase 3, chlorophyll a/b binding protein of LHCII type III chloroplast precursor, light-harvesting complex 1 and S-adenosylmethionine synthetase (Karuna Sree et al., 2000; Inoue et al., 2003; Finkelstein, 2013; Liu et al., 2013; Zhai et al., 2013; Kim et al., 2015; Saruhashi et al., 2015). The expression of some of these genes could potentially lead to significant protective activity in wheat. For instance, aquaporins (AQPs) are membrane proteins found in a large variety of organisms, mainly known for their role in water transport. As described by Tian et al. (2016), a member of AQP subfamily PIP1, AtPIP1;4, found in *Arabidopsis thaliana*, has been shown to play a central role in pathogen associated-molecular patterns (PAMPs)-triggered immunity (PTI) and SAR in the plant, by regulating H_2_0_2_ transport across the plasma membrane, from the apoplast to the cytoplasm, hence playing an active role in triggering H_2_0_2_-dependent host defenses. Another AQP, AtPIP1;2, has been reported to be involved in PTI and ABA-dependent stomatal closure in *A. thaliana* (Rodrigues et al., 2017). Additionally, genes involved in carbohydrate metabolism were over-transcribed in the priming modality. ABA has been described as primordial in the regulation of carbohydrate metabolism during plant stresses (Kempa et al., 2008). Another identified relevant DEG in the priming modality is *ABC1-family like*. Indeed, Wang et al. (2013) have recently reported that *TaAbc1* upregulation is associated with hypersensitive response against stripe rust in wheat. We also found an overexpression of DEGs involved in amino acid, protein, lipid and energy metabolism, that could be regulated by mycosubtilin to compensate the potential deleterious effect of *Z. tritici* infection on wheat metabolism. Finally, four DEGs associated with signaling, transcription and translation were upregulated in the priming modality, highlighting the significant broader amplitude of responses displayed in mycosubtilin-primed and infected wheat plants than in naïve infected plants.

### 4.4 Mycosubtilin did not elicit marked metabolite regulation but primes flavonoid accumulation in wheat leaves

Metabolomic analyses showed a substantially higher number of DAMs in the priming modality than in the elicitation ones, thus agreeing with transcriptomic findings. Indeed, only a few metabolites were regulated upon treatment with mycosubtilin in the elicitation modality, such as methyl jasmonate (MeJA) at D5. At D15, a flavonoid (tricin) was found under-accumulated, whereas four metabolites were detected in higher concentrations, *i.e* glutamic acid, threonine as well as, remarkably, ABA glucose ester (ABA-Glc), and proline. Proline is an osmolyte amino acid involved in adaptation to abiotic stress, especially salt, osmotic, and drought stresses (Szabados and Savouré, 2010). Proline accumulation is supposed to be regulated by ABA (Finkelstein, 2013). Although the concentration of ABA and derivatives was not significantly regulated in treated wheat leaves, except at D15 for ABA-Glc, many DEGs and DAMs associated to ABA-dependent pathways were scored, suggesting that wheat responses to mycosubtilin may involve this hormone (both in elicitation and priming modalities). Whereas the role of ABA in plant tolerance to abiotic stress, such as drought, salinity, cold, and heavy metals has been extensively investigated, its effect on plant pathogen resistance remains unclear (Asselbergh et al., 2008; Vishwakarma et al., 2017). As reviewed by Lievens et al. (2017), a negative effect of this phytohormone on plant resistance has been reported for many pathosystems, such as tomato*-B. cinerea* and rice*-Magnaporthe oryzae*. In the rice-*M. grisea* pathosystem, ABA has been shown to compromise rice resistance against the pathogen by acting antagonistically towards SA-dependent pathway (Jiang et al., 2010). Some phytopathogens were even able to biosynthesize their own ABA, such as *B. cinerea* and *M. oryzae*, most likely to modulate host defenses and enhance their pathogenicity (Ding et al., 2015; Spence et al., 2015). However, in the later years, this phytohormone has also been described to display positive effects on plant resistance in other pathosystems, such as *A. thaliana*-*Pythium irregulare* and *Brassica napus*-*Leptosphaeria maculans* (Adie et al., 2007b; Kaliff et al., 2007). Two ABA-dependent modes of action on plant resistance against pathogens have been highlighted, including the regulation of stomatal closure to prevent pathogens from invading the plant, and callose deposition (Ton et al., 2009). In addition, ABA has also recently been proposed as a potential key actor in molecular cross-talks for plant-microbe symbiosis at the rhizosphere level (Stec et al., 2016). In the wheat-*Z. tritici* pathosystem, the role of ABA, as well as ethylene, in the host resistance mechanisms has, so far, been clearly overlooked. Hence, further investigations focusing on this phytohormone may provide new insights on its role in wheat resistance against *Z. tritici*.

In the priming modality at D5, only an under-accumulation of metabolites was observed, with patterns of amino acids and benzoxazinoids in opposition to those scored in the fungal effect modality at the same time point. This result suggests that mycosubtilin treatment (weakening the fungus pathogenicity) may reduce the expected fungal effect on benzoxazinoid and amino acid pathways in wheat, likely due (i) to its direct antifungal activity and/or (ii) to a countering by the plant of *Z. tritici* effect on these two pathways thanks to a compensatory mechanism. However, at D15, we observed significant changes in metabolite accumulation. The most remarkable result was clearly the increased accumulation of almost all targeted flavonoids, especially luteolin-derivatives (**Figures 6**, **7**). Noticeably, at D5 in the priming modality, the genes encoding chalcone synthase 1 and 2, enzymes involved in flavonoid biosynthesis, were significantly upregulated (**Figure 7**). Flavonoids are known to exhibit a range of biological activities in plant protection, not only antimicrobial activity but also strong antioxidant activity and cell-wall reinforcing (Lam et al., 2015; Lan et al., 2015; Al Aboody and Mickymaray, 2020). Accumulation of compounds displaying these three activities could be greatly beneficial for the host, especially just at the moment preceding the *Z. tritici* switch to necrotrophic phase. Indeed, at this time, *Z. tritici* growth increases, and the fungus produces toxins to induce wheat cell apoptosis, in order to feed on the released nutrients (Steinberg, 2015). Hence, cell-wall strengthening and the increase in antioxidant capacity may very likely help wheat mesophyll cells to resist to the pathogen attack. On the other hand, hydroxycinnamic acid amides (coumaroylputrescine, coumaroylcadaverine, and caffeoylputrescine) were significantly under-accumulated. The decrease in the concentration of these metabolites in wheat leaves may be linked with the hypotheses regarding mycosubtilin effect on plant metabolome during the priming modality emitted above (due to direct mycosubtilin antifungal effect and/or plant compensatory mechanism). Nevertheless, another reason would be the reorganization of metabolic pathways due to the mycosubtilin priming effect, as illustrated in **Figure 7**. Hence, during infection, flavonoid biosynthesis pathway would be enhanced to the detriment of the hydroxycinnamic acid amide pathway, leading to this differential accumulation in wheat leaves in the priming modalities. Interestingly, flavonoid biosynthesis can be induced by ABA, highlighting once again that ABA-dependent responses may be crucial to understand wheat responses to mycosubtilin (Gai et al., 2020).

In conclusion, this study provides new insights into the mechanisms underlying the bioactivity of the *B. subtilis* lipopeptide mycosubtilin on the wheat-*Z. tritici* pathosystem. This promising biomolecule likely confers protection to wheat through a dual activity, *i.e.* directly by displaying an antimicrobial activity against the fungus, and indirectly by priming the host defense responses. Taken together, these findings reveal that mycosubtilin acts on wheat as a priming agent, likely by involving ABA, and ETH biosynthesis and signaling pathways. Stimulation of the plant immune system by mycosubtilin probably results from the interaction of the biomolecule with the plasma membranes of leaf cells leading to the activation of abiotic stress-like responses in the plant. However, further investigations are required to unravel the precise mechanisms by which wheat leaf cells perceive mycosubtilin within the membranes.

## Data availability statement

The datasets presented in this study can be found in online repositories. The names of the repository/repositories and accession number(s) can be found in the article/**Supplementary Material**.

## Author contributions

All authors listed have made a substantial, direct, and intellectual contribution to the work and approved it for publication.
