# Supplementary material for: Deciphering immune responses primed by a bacterial lipopeptide in wheat towards Zymoseptoria tritici
Source: Front Plant Sci. 2023 Jan 26;13:1074447. doi: 10.3389/fpls.2022.1074447 (PMC9909289; doi:10.3389/fpls.2022.1074447)
Supplement: Supplementary file 1 [file DataSheet_1.docx]

Supplementary Material

# Supplementary Figures


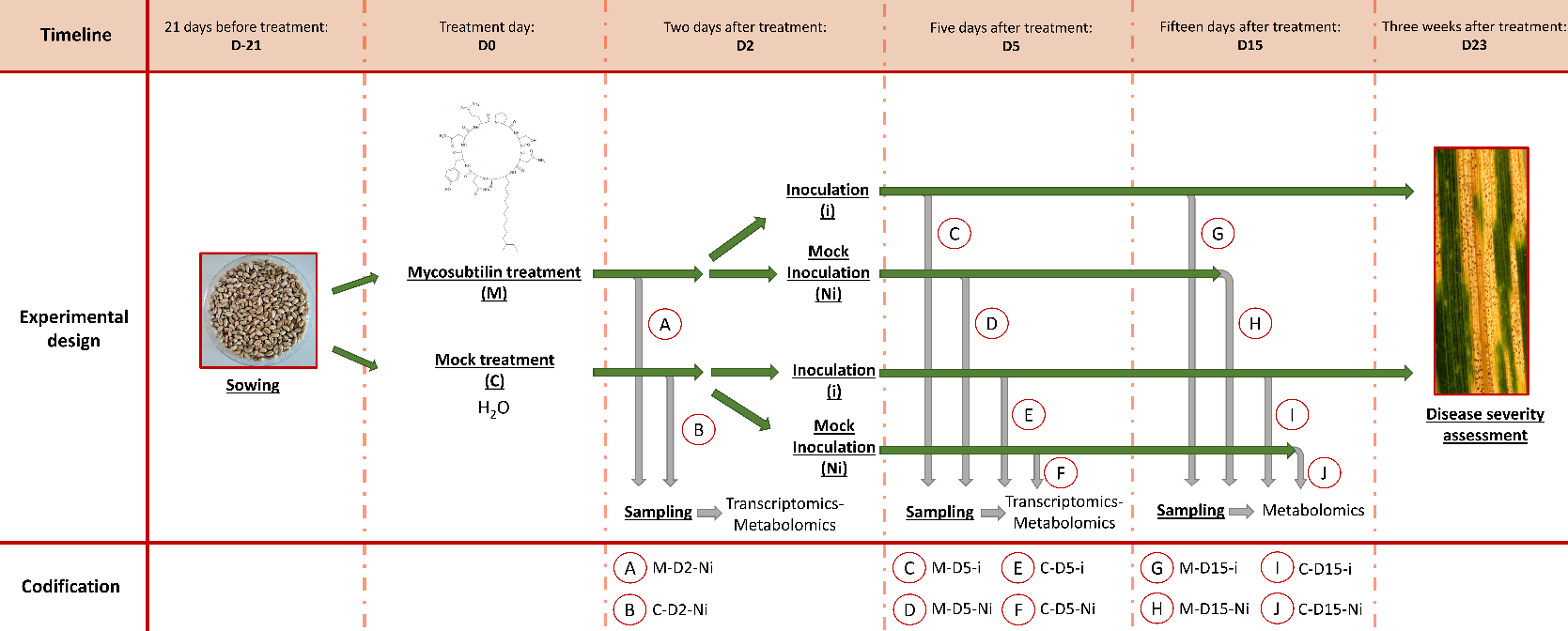


**Supplementary Figure S1.** Experimental design illustrating the main steps of the whole study. After pre-germination in Petri dishes, wheat seeds (cv. Alixan) were grown in greenhouse for 21 days. At days 0 (D0), plants were either sprayed with a solution of mycosubtilin at 100 mg.L^-1^ or with a mock treatment. Photography of the epidermis of wheat third-leaves observed with an optic microscope is shown. At two days after treatment (D2), third leaves were harvested for each condition and were used for further analyses (transcriptomics and metabolomics). After that, remaining plants were inoculated, or not, with a spore suspension of the *Zymoseptoria tritici* strain T02596 (concentration: 10^6^ spores mL^-1^). Photography of *Z. tritici* growing on PDA medium and observed microscopically is presented. At five days (D5) and fifteen days (D15) after the treatment, wheat third leaves were sampled for further analyses; assessment of the *in planta* infection process of the fungus at D5 was investigated as well as transcriptomic analyses whereas metabolomic assays were performed at D5 and D15. Finally, at 23 days after treatment (D23), disease severity level was scored by measuring the third-leaf area with disease lesions. Codification used for each modality in the present study is shown at the bottom of the figure.


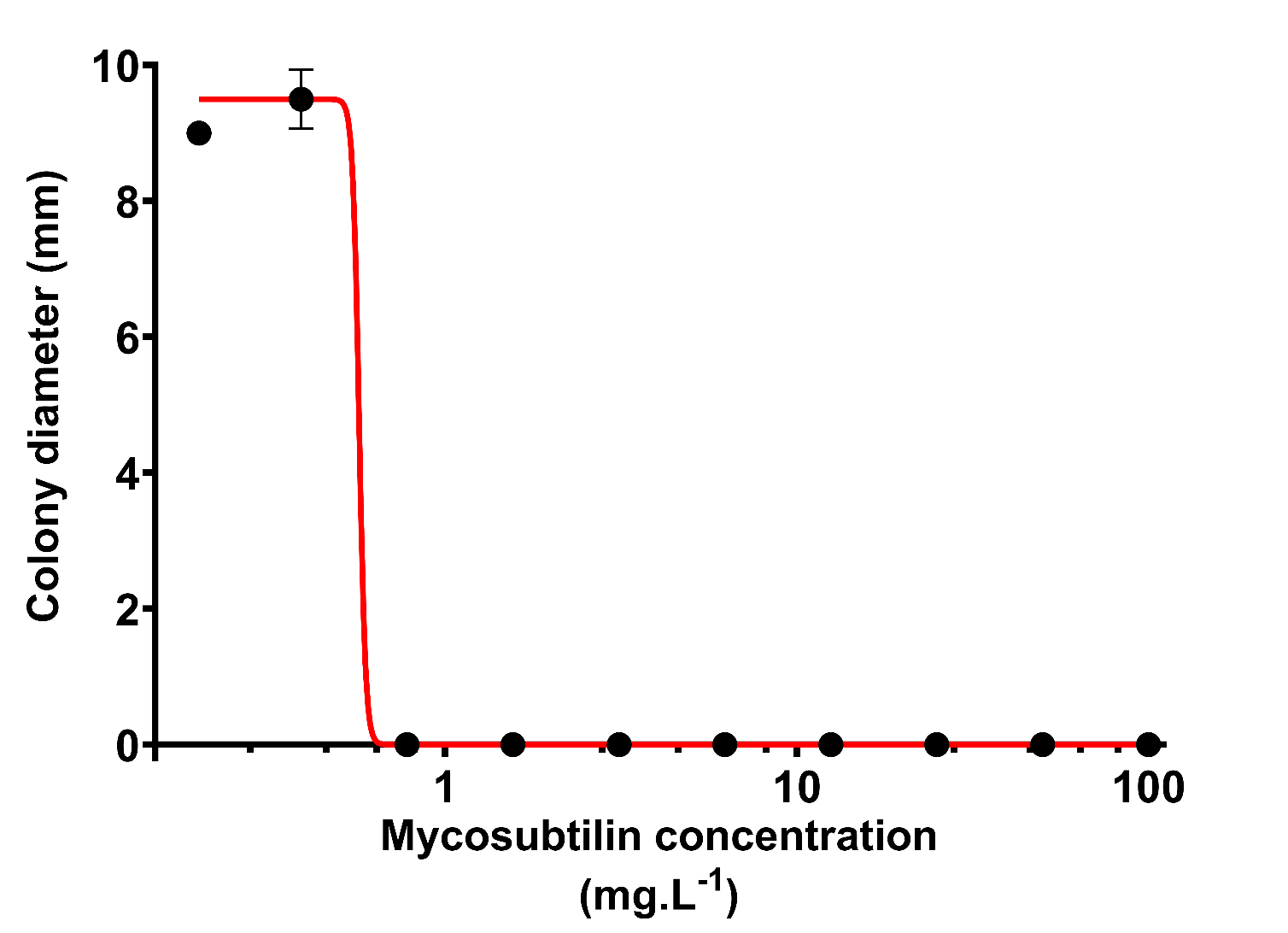


**Supplementary Figure S2.** Dose-response curve obtained *in vitro* for mycosubtilin towards *Zymoseptoria tritici* strain T02596. The fungal growth was assessed on PDA medium amended or not with different concentrations of mycosubtilin and scored 10 days of incubation by measuring the perpendicular diameters of the fungal colonies (n=6 colonies)**.** Non-linear regression was performed and IC_50_ was determined at 0.57 mg.L^-1^ using GraphPad Prism software version 9.


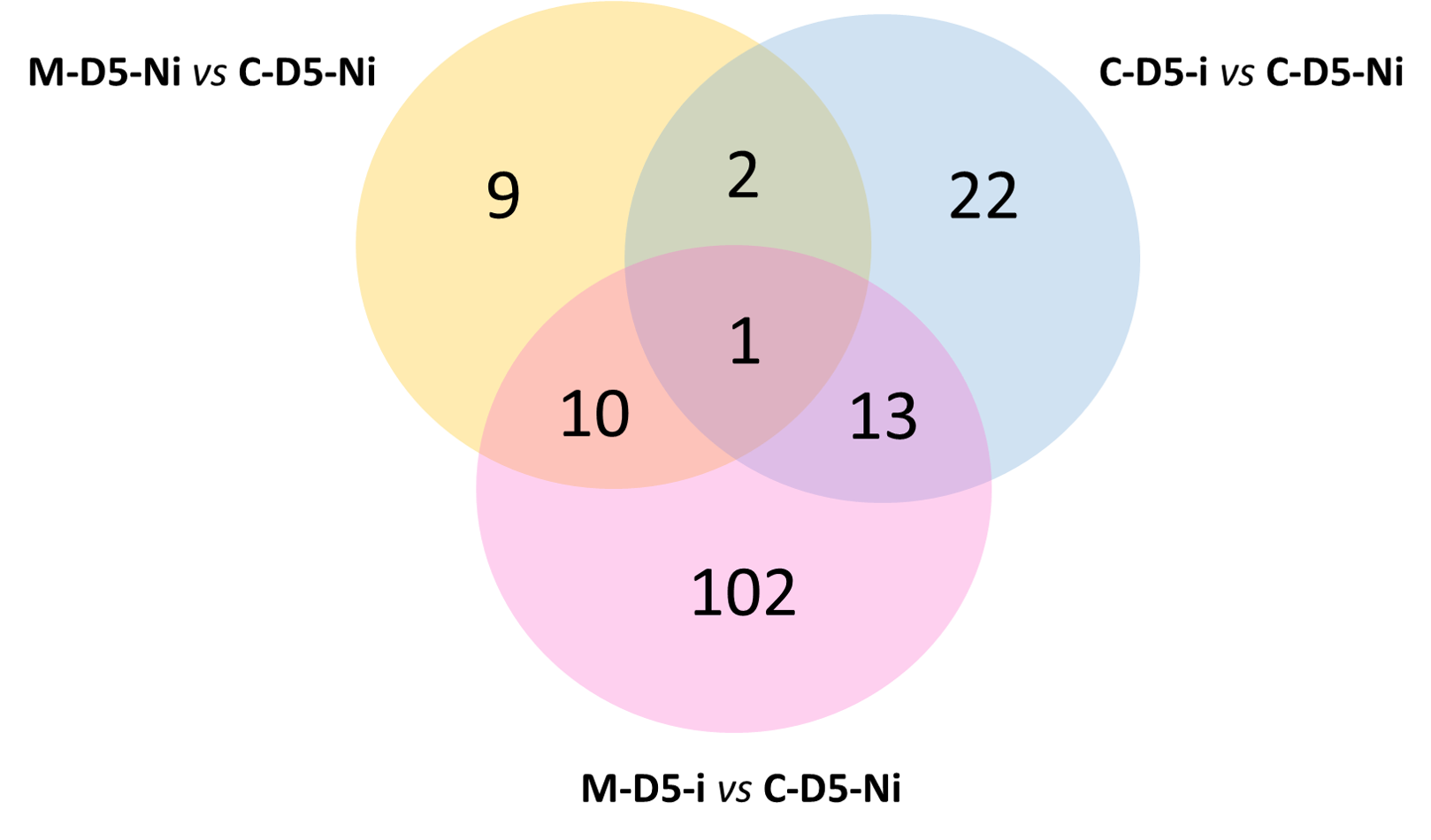


**Supplementary Figure S3.** Venn diagram of the number of differentially expressed genes observed when investigating the eliciting effect of mycosubtilin (M-D5-Ni *vs* C-D5-Ni), *Zymoseptoria tritici* effect (C-D5-i vs C-D5-Ni) and the combined effect of both mycosubtilin and *Z. tritici* (M-D5-i vs M-D5-Ni) at five days after treatment with mycosubtilin (*i.e.* three days after wheat inoculation with *Z. tritici*). M means treated with mycosubtilin towards mock treated C. Ni stands for mock inoculated whereas i indicates that plants were infected with *Z. tritici*. D5 mean that leaves were sampled at five days after mycosubtilin treatment.


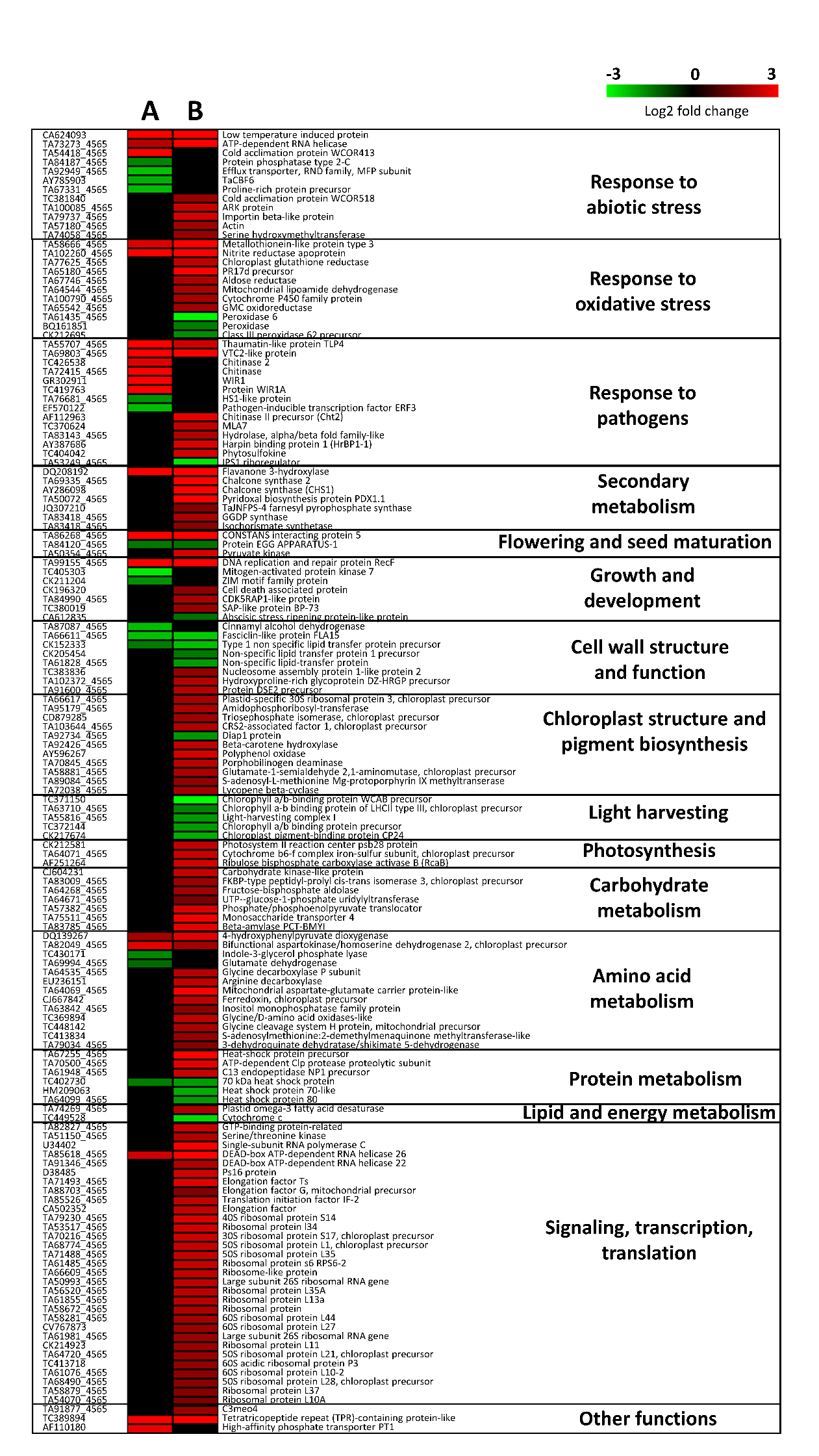


**Supplementary Figure S4.** Heatmap of differentially expressed genes in wheat leaves (Cv.Alixan) after *Zymoseptoria tritici* (T02596 strain) infection in presence of mycosubtilin, at five days after treatment (*i.e.* three days after inoculation). (A) Comparison of gene expression between wheat leaves treated with mycosubtilin and inoculated with the fungus *versus* leaves treated with mycosubtilin and non-inoculated (M-D5-i *vs* M-D5-Ni). (B) whole specter of gene regulation induced by the combined effect of both mycosubtilin and *Z. tritici* (MD5i *vs* CD5Ni). Gene-related physiological processes are represented on the right part of the graph and were determined using NCBI, AmiGO 2 Gene Ontology, KEGG and UniProt. Significant relative changes in gene expression are presented in Log2 ratio, according to the corresponding color scale, using the WebMev software. M means treated with mycosubtilin towards mock treated C. Ni stands for mock inoculated whereas i indicates that plants were infected with *Z. tritici*. D5 means that leaves were sampled at five days after mycosubtilin treatment.


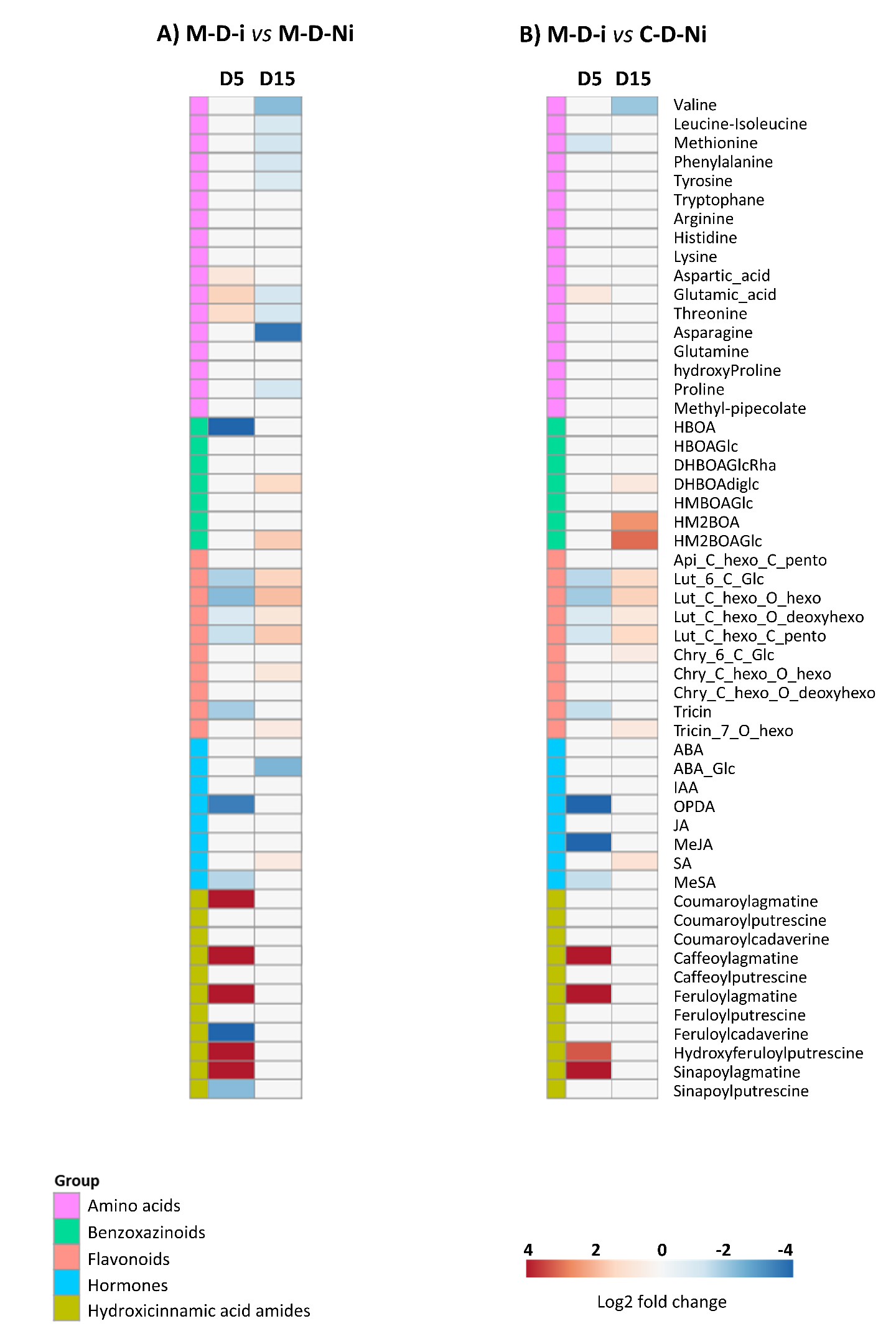


**Supplementary Figure S5.** Heatmap of significant relative changes in metabolite pattern accumulation within wheat third-leaves (Cv. Alixan) following mycosubtilin application and/or *Zymoseptoria tritici* (T02596 strain) infection, at different time points (n=9 plants). (A) From the left to the right, the following conditions are presented, M-D5-i *vs* M-D5-Ni and M-D15-i *vs* M-D15-Ni. (B) Whole specter of metabolite accumulation modifications induced by the combined effect of mycosubtilin treatment and *Z. tritici* infection (from the left to the right M-D5-i *vs* C-D5-Ni and M-D15-i *vs* C-D15-Ni). M means treated with mycosubtilin towards mock treated C. Ni stands for mock inoculated whereas i indicates that plants were infected with *Z. tritici*. D5 and D15 mean that leaves were sampled respectively at five and fifteen days after mycosubtilin treatment. Log2 of significant metabolite fold changes for indicated pairwise comparisons are given by shades of red or blue colors according to the scale bar. Metabolites were grouped according to their functional or chemical family as amino acids, benzoxazinoids, flavonoids, hormones and hydroxycinnamic acid amides. Data represent mean values of nine biological replicates for each condition and time point. Statistical analyses were performed using the Tukey's Honest Significant Difference method followed by a false discovery rate (FDR) correction, with FDR < 0.05. For FDR ≥ 0.05, Log2 fold changes were set to 0.


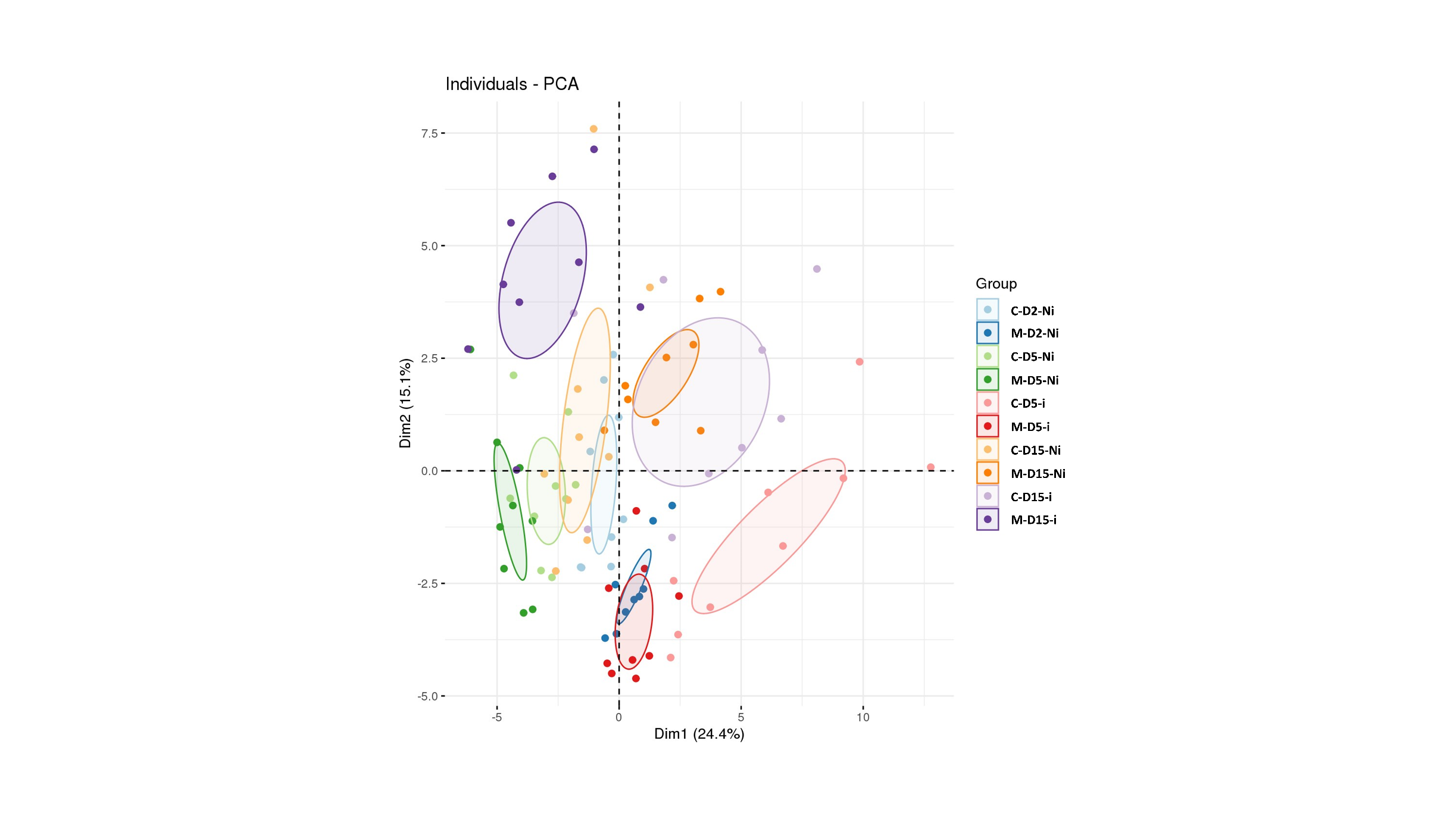


**Supplementary Figure S6.** Principal component analysis (PCA) displaying the global impact of mycosubtilin treatment and infection with *Zymoseptoria tritici* on wheat leaf metabolome. PCA was performed on relative amounts of all analyzed compounds in the different groups of samples. For each group, nine biological replicates were used. The first two principal components explain 24.4% and 15.1% of the variance separating the groups of samples, respectively.
